# New Insights Into the Anticonvulsant Effects of Essential Oil From *Melissa officinalis* L. (Lemon Balm)

**DOI:** 10.3389/fphar.2021.760674

**Published:** 2021-10-14

**Authors:** Ben A. Chindo, Melanie-Jayne R. Howes, Sawsan Abuhamdah, Musa I. Yakubu, Godwin I. Ayuba, Alex Battison, Paul L. Chazot

**Affiliations:** ^1^ Department of Pharmacology and Toxicology, Faculty of Pharmaceutical Sciences, Kaduna State University, Kaduna, Nigeria; ^2^ Royal Botanic Gardens Kew, Richmond, United Kingdom; ^3^ Department of Biosciences, Durham University, Durham, United Kingdom; ^4^ College of Pharmacy, Al Ain University, Abu Dhabi, United Arab Emirates; ^5^ Department of Biopharmaceutics and Clinical Pharmacy, Faculty of Pharmacy, University of Jordan, Amman, Jordan; ^6^ Department of Anatomic Pathology and Forensic Medicine, College of Medicine, Kaduna State University, Kaduna, Nigeria

**Keywords:** *Melissa officinalis*, essential oil, epilepsy, 4-AP brain slice model of epilepsy, sustained repetitive firing, voltage-gated sodium channels, kindling

## Abstract

*Melissa officinalis* L. is used in traditional European and Iranian folk medicines to treat a plethora of neurological diseases including epilepsy. We utilized the *in vitro* and *in vivo* models of epilepsy to probe the anticonvulsant potentials of essential oil from *M. officinalis* (MO) to gain insight into the scientific basis for its applications in traditional medicine for the management of convulsive disorders. MO was evaluated for effects on maximal electroshock (MES) and pentylenetetrazole (PTZ) -induced seizures in mice, on 4–aminopyridine (4-AP)-brain slice model of epilepsy and sustained repetitive firing of current clamped neurons; and its ameliorative effects were examined on seizure severity, anxiety, depression, cognitive dysfunction, oxidative stress and neuronal cell loss in PTZ-kindled rats. MO reversibly blocked spontaneous ictal-like discharges in the 4-AP-brain slice model of epilepsy and secondary spikes from sustained repetitive firing, suggesting anticonvulsant effects and voltage-gated sodium channel blockade. MO protected mice from PTZ– and MES–induced seizures and mortality, and ameliorated seizure severity, fear-avoidance, depressive-like behavior, cognitive deficits, oxidative stress and neuronal cell loss in PTZ–kindled rats. The findings warrant further study for the potential use of MO and/or its constituent(s) as adjunctive therapy for epileptic patients.

## Introduction

Epilepsy is a progressive and chronic neurological disorder characterized by unprovoked spontaneous recurrent seizures (SRS), ictal and inter-ictal events or generally asymptomatic bouts of population level activities that are measured between seizures. It is associated with profound physical, social and psychological consequences, and without age, racial, social, sexual or geographical boundaries (Gidal et al., 2002).

This multifaceted neurological disorder that affects 1–3% of the world’s population (Loscher, 2002) may be idiopathic, and the underlying cause is unknown except for a possible genetic predisposition. In many patients with unexplained epilepsy, the generally accepted theory is that epilepsy is the result of an imbalance of certain neurotransmitters and/or ion channels that regulate membrane excitability. Neurotransmitters such as acetylcholine, aspartate, corticotrophin-releasing factor, cytokines, glutamate, histamine, noradrenaline, peptides, purines and steroid hormones enhance excitability and propagation of neuronal activity, whereas dopamine and γ-aminobutyric acid (GABA) inhibit neuronal activity and propagation (Rogers and Cavazos, 2008).

It has also been shown that epilepsy can result from infectious, metabolic, malformative, traumatic, tumoral or vascular conditions (Engel, 2001). Diseases such as Alzheimer’s disease (AD), cerebral malaria (CM), bacterial meningitis, trauma and brain infections are common causes of epilepsy (Murthy and Prabhakar, 2008; Ngoungou and Preux 2008; Miranda and Brucki, 2014). AD carries a significantly increased risk of developing epilepsy and seizures (Miranda and Brucki, 2014), and it has been shown that people with hereditary/early-onset AD have developed unprovoked seizures at some point. Cerebral malaria is a potential cause of epilepsy in most of sub-Saharan Africa, which is considered an endemic area of the world (Ngoungou and Preux, 2008; Dubé et al., 2009). In developing countries, an increased frequency of bacterial meningitis has been associated in part with a high incidence and prevalence of epilepsy (Murthy and Prabhakar, 2008). Studies have demonstrated a relationship between epilepsy and bacterial meningitis (Rantakallio et al., 1986; Annegers et al., 1988; Bedford et al., 2001) and this debilitating neurological disorder has been shown to result from long-term bacterial meningitis (Murthy and Prabhakar, 2008).

Although non-pharmacological therapies are important in disease management, pharmacological intervention remains the “Gold standard” in the treatment of epilepsy despite the deleterious effects and drawbacks of currently available antiepileptic drugs (AEDs) (Errington et al., 2005). Conventional AEDs have failed to either provide a cure for epilepsy or prevent relapse from epileptic attacks and their use is often associated with serious untoward reactions (Chindo et al., 2009). Furthermore, these therapeutics are effective in only about 30–40% of epilepic patients. The continued search for new therapeutics to surmount these impediments has become a pertinent venture in drug management of epilepsy.

*Melissa officinalis* L. (lemon balm) in the family Lamiaceae, is a perennial herbaceous medicinal plant with a common native range from the Mediterranean to Central Asia, which has also been introduced to Europe and the Americas. It is widely cultivated as a culinary and medicinal herb (Meftahizade et al., 2010) and its preparations are used in traditional European and Iranian folk medicines for management of gastrointestinal disorders, anxiety, pain, stress induced headache, insomnia, epilepsy, depression, psychosis and hysteria (Blumenthal et al., 2000; Shakeri et al., 2016). Previous studies have shown that *M. officinalis* has anti-tumor and anti-viral effects (Janina, 2003; Turhan, 2006) with potential relevance for management of AD (Howes and Houghton, 2012) and epilepsy (Bhat et al., 2012; Ghayour et al., 2012), while the essential oil in particular, shows neuroactive effects (Elliott et al., 2007; Abuhamdah et al., 2008). In this study, we use *in vitro* and *in vivo* models of epilepsy to explore the anticonvulsant potential of *M. officinalis* essential oil (MO) to understand the scientific basis for using this species in traditional medicine to treat convulsive diseases.

## Materials and Methods

### Animals

Adult Swiss mice (20–25 g) and Wister rats (180–200 g) of 8–9 weeks old obtained from Animal Facility Centre (AFC) of Kaduna State University (KASU) were used in this study, were. The mice and rats were housed in groups of five in transparent plastic cages under standard conditions of temperature (22 ± 1°C), relative humidity and light/dark cycles (12/12 h). The animals were fed with Vital feeds and water ad libitum. We minimised the number of animals used and their suffering in this study. *In vivo* experiments were performed from 8:00 am to 3:00 pm, and in accordance with the National Institute of Health Guidelines for the Care and Use of Laboratory Animals (NIH Publications No. 80-23) revised 1996. *In vitro* experiments were conducted under United Kingdom Home Office guidelines.

### Gas Chromatography-Mass Spectrometry (GC-MS) Analysis

*Melissa officinalis* essential oil (MO) obtained from G. Baldwin & Co (London SE17 1RW) was subjected to GC-MS analysis using an Agilent 7890A GC coupled to an Agilent 5975C MS. Using an oven program of 40–220°C at 3°C/min Chromatography was performed on a 30 m × 0.25 mm ID × 0.25 µm DB-5MS column (J. & W. Scientific, United States). The carrier gas was helium at a flow rate of 1 ml/min and 1 µl injections (split 1:10) of the oil (diluted 1/100 in diethyl ether; GLC pesticide residue grade, Fisher, United Kingdom) at 220°C were made by an autosampler. Detection was by MS, fitted with an Electron ionization (EI) source operated at 70 eV with a source temperature of 180°C, and mass spectra were recorded in the range *m/z* 38–600. Retention indices were calculated against an *n*-alkane series (C10–C16, Supelco, United Kingdom) and compounds were identified by comparing retention indices and/or mass spectra with published data (Adams, 2007; NIST, 2008). Percentage compositions were calculated by integrating all detected peaks in total ion chromatograms (TIC).

### *In vitro* Anticonvulsant Studies

#### Preparation of Brain Slices

Brain slices were prepared from 12 to 16 day old juvenile Sprague–Dawley rats of either sex as described previously (Holopainen, 2008) and modified by Chindo et al. (2009). Briefly, rats were anaesthetized with intraperitoneal injection of pentobarbital sodium and humanely decapitated after loss of righting reflex. Their brains were removed and transferred into cold (0–4°C) sucrose artificial cerebrospinal fluid (aCSF) (mM: sucrose 248, KCl 3, MgCl_2_ 1, CaCl_2_ 1, NaH_2_PO_4_ 1.25, NaHCO_3_ 26, and glucose 10) bubbled with carbogen. Sagittal slices (650 µm) with both hippocampus and visual cortex were prepared using a vibratome in a slicing chamber with bubbled sucrose aCSF. The slices were rapidly transferred into a holding chamber containing bubbled standard aCSF (mM: NaCl 124, KCl 3, MgCl_2_ 1, CaCl_2_ 2, NaH_2_PO_4_ 1.25, NaHCO_3_ 26, and glucose 10) and placed in a water-bath at 37°C for 30–60 min. The slices were then stored at room temperature prior to use.

#### Brain Slice Extracellular Recordings

Brain slices perfused with oxygenated aCSF at 4 ml/min were transferred into a recording chamber. The solutions were pre-heated in a water bath and using a temperature controller (TC-202A, Harvard Medical Instruments, United States) to maintain the temperature at 34 ± 0.2°C. To induce epileptiform activity, brain slice was perfused with standard aCSF containing 100 µM 4-aminopyridine (4-AP, Sigma Chemical Co.) (Lees et al., 2006) and a microelectrode (1–2 M) filled with 3 M NaCl was placed caudal to the CA1 region of the hippocampus and deep into layers (V/VI) of the visual cortex. Recordings were made after standard aCSF containing the 4-AP was perfused for at least 45 min. A brain slice was used for experiments if discharge intervals over three to four events was obtain. If no ictal activity was observed where two consecutive events supposed to occur, effects of the drugs were recorded as complete block. The oil was solubilized in 0.5% tween80 in normal saline and the 4-AP was formulated daily in bubbled aCSF; and both were applied through Teflon coated tubing.

Data were recorded with a preamplifier (World Precision Instruments, United States) model 767-B connected to a differential amplifier in series to multiply the signal (100× to levels). The analog signals were digitalized at 6 kHz on-line using a CED Micro 1401 MKII (Cambridge Electronic Design, United Kingdom), and data were filtered at 3 kHz to Oscilloscope (OX7520, ITT Instruments, National Instruments, United States). Ictal events were scored using wavemark template (Spike 2 version 6).

#### Cell Culture Techniques

Seventeen to eighteen-day old embryos were collected from donor Sprague–Dawley rats for dissection as described by Lees and Leach (1993). Rats were humanely euthanised with pentobarbital sodium, brains from embryos removed with sterilized forceps, triturated with Pasteur pipettes and dissociated cells (200,000 cells/ml) plated on poly-d-lysine coated cover slips in Dulbecco’s modified Eagle’s medium supplemented with 100 units/ml penicillin and 100 g/ml streptomycin and 10% foetal calf serum. Twenty four hours later, the plating medium was replaced with a maintenance medium comprising neurobasal medium, with 2% B27 supplement, 100 units/ml penicillin and 100 g/ml streptomycin and 1% glutamax. After 1 week, the cells were fed by carefully replacing 1 ml of medium with 2 ml of freshly prepared maintenance medium, and maintain the cultured cells for at least 3 weeks before being used in electrophysiological studies.

#### Sustained Repetitive Firing (SRF)

Cells were current clamped at their resting membrane potentials (52.4 ± 0.75 mV, *n* = 5) in extracellular saline (in mM): NaCl 142, KCl 2.5, CaCl_2_ 1, MgCl_2_ 2, CoCl_2_ 2, HEPES 5 and d-glucose 30, pH 7.4 (NaOH 1) supplemented with Cobalt (2 mM), to examine sustained repetitive firing. To activate only the voltage-gated sodium channels (VGSCs), Cobalt (2 mM) was added to extracellular saline to block the voltage-gated Ca^2+^ channels and suppress spontaneous synaptic traffic. SRF was evoked in cells with a resting membrane potential more negative than –50 mV, by applying depolarizing current pulses (750 ms duration at 1 min intervals) through the patch pipette. To produce trains of overshooting action potentials, the pulses were scaled up *via* a DS2A constant voltage isolated stimulator (Digitimer Ltd., United Kingdom). Effects of MO were measured at 10 kHz and filtered at 5 kHz after superfusing MO into the bath for ≥4 min. The number of action potentials in each cell were scored using Wavemark templates (Spike 2 CED, United Kingdom).

### *In vivo* Anticonvulsant Studies

#### Acute Seizures Test

The acute anticonvulsant effects of MO in mice were assessed as described by Bum et al. (2001) and Chindo et al. (2009; 2014) with modifications. Briefly, pentylenetetrazole (PTZ, Carl Roth, Karlsruhe, Germany, 85 mg/kg) was administered (i.p.) 30 min post-administration of the essential oil or phenytoin to induce convulsions (seizures) in mice. An electroconvulsive therapy machine (Ugo Basile, Model 7800) was used to induce seizures through corneal electrodes in the maximal electroshock seizure (MES) study. The current, impact duration, and frequency used in the entire study are about 150 mA, 0.2 s, and 50 Hz, respectively. The treated groups received *M. officinalis* essential oil (MO, 10–200 mg/kg). MO was solubilized in 0.5% tween80 in normal saline and these doses of MO were selected based on previous studies in our Lab (unpublished data). Phenytoin (Sigma Chemical Co.) was used as the standard anticonvulsant drug. In both the PTZ and MES acute tests, the ability of MO (10–200 mg/kg) or Phenytoin (10 and 25 mg/kg) to prevent or delay tonic hind limb extension or death of mice connotes anticonvulsant activity (Chindo et al., 2009).

### PTZ Chronic Kindling Studies

A sub-effective dose of pentylenetetrazole (PTZ, Carl Roth, Karlsruhe, Germany). The subeffective dose of PTZ (35 mg/kg, i.p.) was established in a preceding experiment. A total of 16 subconvulsive injections of PTZ were administered on alternate days and control animals received normal saline injections. Thirty minutes prior to PTZ, the treatment and saline groups concurrently received MO (50 and 100 mg/kg, i.p.) solubilized in 0.5% tween80 in normal saline. The seizure severity were observed and classified for 20 min after each PTZ injection using a modified Racine scale (Becker et al., 1995) as follows:

Stage 0: no response;
Stage 1: facial and ear twitching;
Stage 2: myoclonic jerks without rearing;
Stage 3: rearing, myoclonic jerks;
Stage 4: clonic-tonic seizures, turning over into side position;
Stage 5: generalized clonic-tonic seizures, turning over into back position.

The ameliorative effects of the essential oil on anxiety, depression and memory deficits in the kindled rats were evaluated using established behavioral models, 24 hours after kindling completion. One week after the last PTZ kindling injection, the rats received a challenge dose of PTZ (32.5 mg/kg, i.p.) and resultant convulsive behavior were scored by the modified Racine scale, in order to check the persistence of enhanced susceptibility to the PTZ.

### Behavioral Assessment

#### Open Field Test (OFT)

The open field is made up of white plywood (120 cm × 120 cm × 50 cm), the floor divided with visible lines into 36 (20 cm × 20 cm) squares, a central square (20 cm × 20 cm) drawn in the middle (Brown et al., 1999) and lighting set at ∼90 lux in the open field. Each rat was placed in the open field and allowed to explore the apparatus for 5 min. Line crossing, rearing counts, grooming time, freezing duration, centre square entries and time spent in the centre square, defecation and urination were recorded (Rogoz et al., 2003; Santosh et al., 2011). The apparatus was cleaned and dried with 70% ethanol between each sessions (Lindholm et al., 2012).

#### Elevated Plus Maze (EPM)

The elevated plus maze, a widely used apparatus to assess situational anxiety states in rodents (Salzberg et al., 2007; Jones et al., 2008) consists of a plus-shaped platform with two opposing arms (50 cm × 10 cm) enclosed by 40-cm high side and end walls (closed arms), and the other two had no walls (open arms) with an opened central square (10 cm × 10 cm) in the middle of the maze, and lighting at this point was set at ∼90 lux. Each rat is placed facing a closed arm in the central area of the maze elevated 50 cm from the floor. The animal was allowed to freely explore the maze for 5 min and its movement video-tracked. During the 5-min exposure, number of entries into open arms, number of entries into closed arms, time spent on the open arms and time spent in centre square were recorded (Cotella et al., 2009). The maze was cleaned and dried with 70% ethanol after each session.

#### Novel Object Recognition Test (NORT)

Cognitive deficit in pentylenetetrazole-kindled rats was assessed using NORT. It is an open box made of Plexiglas (52 cm × 52 cm × 31 cm) and positioned on a moveable trolley 27 cm above the floor. A day before the test, each rat was familiarized with the apparatus for 5 min, and on the first day of the test, two identical objects were presented to each rat to explore for a 5 min. An animal was said to have explored an object when it directs its nose at a distance less than 2 cm to the object or it touches the object. On the second day of the test, one of the objects presented in the first day was replaced with a new object and the time spent by the animal to explore the new object (tB) and the familiar (tA) objects was recorded for 5 min (Kulkarni et al., 2011). The discrimination index (DI) was calculated as (tB/(tB + tA) (Ennaceur and Delacour, 1988; Rajendran et al., 2014).

#### Forced Swim Test (FST)

The forced swim test (FST) as described in previous studies (Porsolt et al., 1978; Detke et al., 1995) was carried out on the PTZ-kindled rats. Each rat on the first day was forced to swim for 15 min (pretest session) in a transparent glass cylinder (height 46 cm, diameter 20 cm) filled with water to a depth of 30 cm at 25°C. The rats were removed from the water after the 15 min pretest, allowed to dry in a heated enclosure (32°C) and returned to their home cages. During the test session on day-two, each rat was placed in the water again and allowed to swim for 6 min. The immobility time was recorded for a period of 5 min (Kroczka et al., 2001) after discarding activity in the first 1 min, with an assumption that an animal would try to escape as soon as it is placed in the water (Jain et al., 2003). A rat was considered immobile when it is floating passively, remained motionless or making slight movements (with one hind limb or the tail) just to keep its head above the water (Chindo et al., 2012).

### Biochemical Assays

#### Tissue Preparation

Rats were anesthetized with ether 24 hours after challenged experiment and humanely decapitated. Whole brains were removed, rinsed with cold 0.9% saline and weighed. The brain samples were homogenized in 10% w/v ice-cold 0.1 M phosphate buffer (pH 7.4), centrifuged at 10, 000 × *g* force at 4°C for 10 min and the supernatants were used for the biochemical studies.

#### Estimation of Glutathione (GSH) Concentration

The concentration of GSH was determined as described previously by Moron et al. (1979) with modifications. Briefly, 0.4 ml of 20% trichloroacetic acid (TCA) was added to 0.4 ml of the brain supernatant, gently mixed by a swirling motion and centrifuged at 10,000 × *g* force for 20 min at 4°C. Two milliliter (2 ml) of 0.6 mM 5, 5–dithiobis-(2-nitrobenzoic acid) (DTNB) was added to 0.25 ml of the supernatant and the volume made up to 3 ml with 0.75 ml phosphate buffer (0.2 M, pH 8.0). A relatively stable (yellow) chromophoric product obtained from the reaction of DTNB with the reduced glutathione (2-nitro-5-thiobenzoic acid). Absorbance was then read at 412 nm against blank reagent [2 ml of 0.6 mM DTNB + 1 ml phosphate buffer (0.2 M, pH 8.0)] using a spectrophotometer. The concentration of reduced GSH in the brain tissues, which is proportional to the absorbance at this wavelength, is expressed as microgram per gram tissue (µg/g tissue).

#### Estimation of Malondialdehyde (MDA) Concentration

The MDA level, which is an index of lipid peroxidation was determined as previously described (Ohkawa et al., 1979). Briefly, Aliquots of 0.5 ml each of 30% TCA and 0.75% thiobarbituric acid (TBA) were added to a mixture of 0.4 ml of the sample and 1.6 ml of Tris-potassium chloride (Tris-KCl) buffer and placed on a water bath for 45 min at 80°C. This was then cooled on ice, and centrifuged at 1,800 ×*g* for 15 min. The pink colour supernatant was collected and absorbance measured against a reference blank of distilled water at 532 nm. The concentration of MDA was expressed as mole of MDA per gram tissue.

#### Estimation of Superoxide Dismutase (SOD) Activity

Brain superoxide dismutase activity was measured as described by Misra and Fridovich (1972) and Benneh et al. (2018). Briefly, 0.75 ml of ethanol (96% v/v) and 0.15 ml of chilled chloroform were added to 0.5 ml of tissue homogenate and centrifuged at 2,000 ×*g* for 20 min at 30 C to obtain a clear supernatant. An aliquot of 0.5 ml of EDTA (0.6 mM) was added to the supernatant solution containing 1 ml of bicarbonate buffer (0.1 M, pH 10.2). To initiate the reaction, 50 μl of adrenaline solution (1.3 mM) was added to the mixture to obtain adrenochrome, the absorbance of which was measured at 480 nm at 25°C. The absorbance of a sample blank containing all reagents apart from tissue homogenate was also measured at 480 nm.

#### Estimation of Catalase (CAT) Activity

The method described by Góth (1991) was employed to determine the brain activity of catalase (CAT). Aliquot of 0.85 ml potassium phosphate buffer 50 mM (pH; 7.0) was added to 0.1 ml of the tissue homogenate and incubated at room temperature for 10 min. To initiate the reaction, 0.05 ml H_2_O_2_ (30 mM prepared in potassium phosphate buffer 50 mM, pH 7.0) was added to the mixture and the absorbance was recorded at 240 nm. Specific activity is expressed as 1 µmol H_2_O_2_ decomposed U/mg protein.

### Histological Studies

The brains were processed for histological examination as previously described by Chindo et al. (2015). Briefly, the rats were anesthetized with ether, rapidly decapitated, the brains were removed and fixed in Bouin’s fluid for 16–24 h, routinely processed and embedded in paraffin. Using rotary microtome, sections of 10 µm thick were cut in the plane of the nucleus habenulae and stained with haematoxylin and eosin. Cells in CA1and CA3 of the rats hippocampus were counted using a counting net in squares of 500 μm × 500 µm. An average of five fields at the left and right hippocampus were counted per rat.

### Statistical Analysis

Data were expressed as mean ± S.E.M. with *n* indicating the number of animals per group for a given experiment. Data were analyzed by one-way analysis of variance (ANOVA) or two-way ANOVA followed by Bonferroni or Turkey *post hoc* test for multiple comparisons using Graph Prism version 4.00 (GraphPad Software, Inc., La Jolla, CA, United States). Results were considered significant at *p* < 0.05.

## Results

### Chemical Composition of *M. officinalis* Oil

The principal constituents detected in the essential oil from *M. officinalis* by GC–MS were monoterpenes and sesquiterpenes (Table 1; GC-MS total ion chromatogram is shown in the Supplementary Figure S1), with composition data consistent with that previously reported for *M. officinalis* oil (Elliott et al., 2007), and with the citral chemotype reported for this species (Kittler et al., 2018a; 2018b).

**TABLE 1:** Percentage composition of assigned compounds detected in the *Melissa officinalis* essential oil (based on all detected peaks), following GC-MS analysis.

Compound	Retention time (min)	*Melissa officinalis* oil percentage composition	RI (experimental)	RI Adams (2007)
α-Pinene	11.7	Tr	906	932
Camphene	12.4	Tr	922	946
Sabinene	13.5	Tr	946	969
β-Pinene	14.0	1.68	959	974
1,8-Dehydro-cineole	14.3	0.29	964	988
Cymene	15.9	Tr	1,001	*o*-: 1,022
				*p*-: 1,020
Limonene	16.1	Tr	1,006	1,024
β-Ocimene	17.0	0.12	1,024	(*Z*)-: 1,032
				(*E*)-: 1,044
Bergamal	17.3	Tr	1,032	1,051
*cis*-Linalool oxide (furanoid)	18.2	Tr	1,051	1,067
*trans*-Linalool oxide (furanoid)	19.2	Tr	1,069	1,084
Linalool	19.7	1.05	1,083	1,095
*cis*-Rose oxide	20.1	0.21	1,093	1,106
*trans*-Rose oxide	20.9	Tr	1,111	1,122
Citronellal	22.2	4.83	1,140	1,148
(*Z*)-Isocitral	22.6	0.39	1,149	1,160
Rosefuran epoxide	23.0	Tr	1,158	1,173
(*E*)-Isocitral	23.5	0.40	1,169	1,169
α-Terpineol	24.3	Tr	1,188	1,186
Neral	26.4	19.54	1,234	1,227
Methyl citronellate	27.2	1.13	1,250	1,257
Geranial	27.8	25.57	1,266	1,264
α-Copaene	32.5	1.42	1,369	1,374
Geranyl acetate	32.6	1.45	1,371	1,379
β-Bourbonene	32.9	0.60	1,377	1,387
β-Elemene	33.0	Tr	1,382	1,389
(*Z*)-Caryophyllene	33.8	0.12	1,397	1,408
(*E*)-Caryophyllene	34.5	11.56	1,413	1,417
β-Copaene	34.8	0.28	1,420	1,430
(*Z*)-β-Farnesene	35.8	0.13	1,441	1,440
α-Humulene	35.9	0.93	1,445	1,452
Allo-aromadendrene	36.1	0.35	1,449	1,458
*trans*-Muurola-4 (14),5-diene	36.5	Tr	1,457	1,465
γ-Muurolene	36.7	0.35	1,462	1,478
Germacrene D	37.0	0.39	1,468	1,484
γ-Amorphene	37.5	Tr	1,478	1,495
α-Muurolene	37.7	0.68	1,484	1,500
(*E*, *E*)-α-Farnesene	37.9	Tr	1,489	1,505
γ-Cadinene	38.3	0.52	1,498	1,513
δ-Cadinene	38.5	0.83	1,502	1,522
*cis*-Calamenene	38.6	0.24	1,505	1,528
α-Cadinene	39.2	0.15	1,518	1,537
α-Calacorene	39.4	Tr	1,523	1,544
β-Calacorene	40.3	Tr	1,542	1,564
Caryophyllene oxide	41.2	13.18	1,561	1,582
Humulene epoxide II	42.2	0.92	1,584	1,608
1-*epi*-Cubenol	42.9	Tr	1,599	1,627
*epi*-α-Cadinol	43.5	0.22	1,612	1,638
*epi*-α-Muurolol	43.6	0.27	1,614	1,640
α-Cadinol	44.0	0.40	1,624	1,652
Calamen-10-ol	44.3	0.08	1,631	*cis-*: 1,660
				*trans*-: 1,668
Germa-4(15),5,10(14)-trien-1-α-ol	46.3	Tr	1,675	1,685
Farnesol	47.6	Tr	1,704	(2*Z*,6*Z*)-: 1,698
				(2*E*,6*Z*)-: 1,714

Tr: < 0.01%.

All compounds were identified by comparing retention indices (calculated against an *n*-alkane series) and mass spectra with published data (Adams, 2007; NIST, 2008), except: compounds that were identified by comparing mass spectra with published data (NIST, 2008).

### *In vitro* Anticonvulsant Studies

**FIGURE 1:**
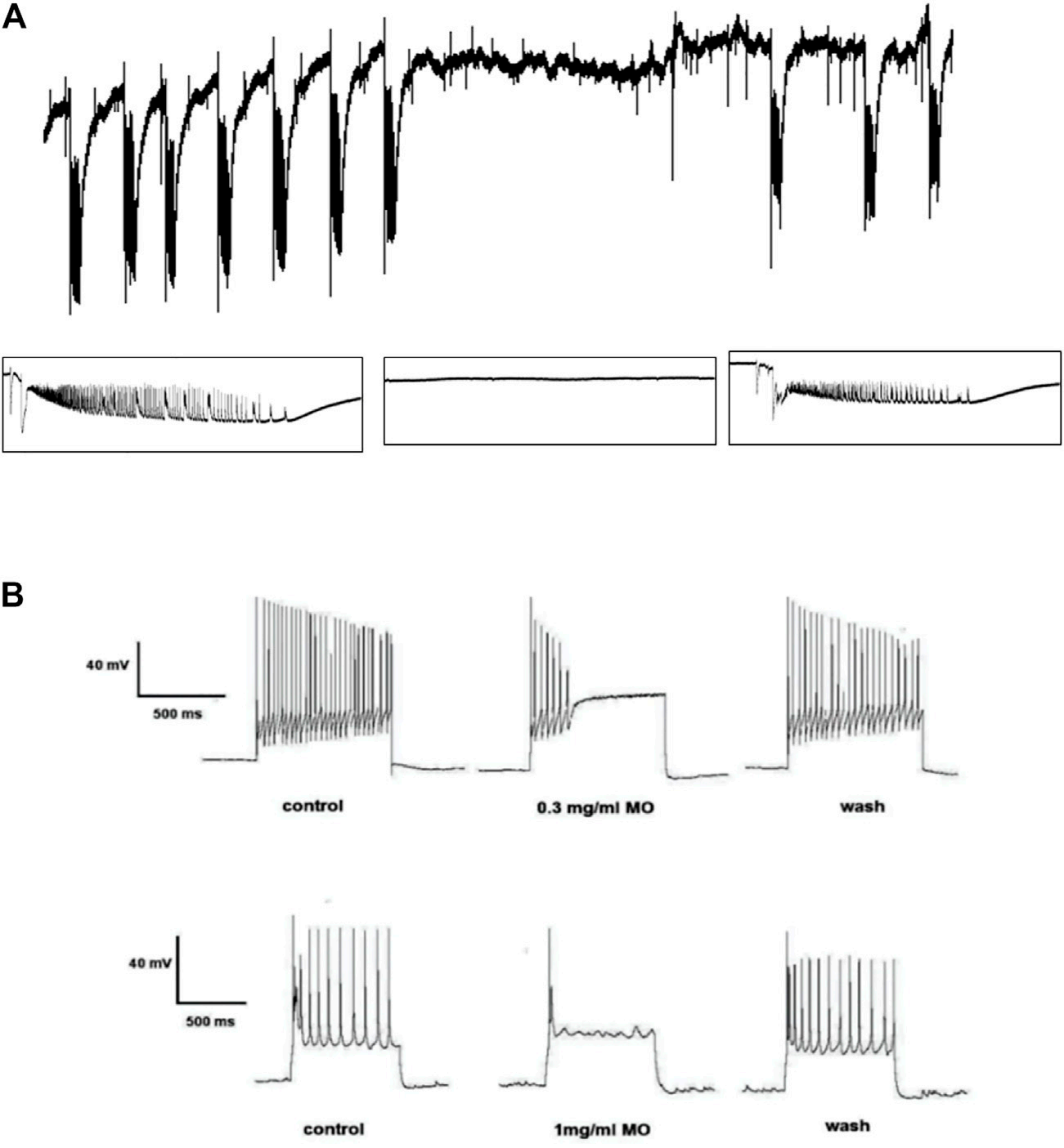
Effects of *Melissa officinalis* L. essential oil (MO) on **(A)** 4-aminopyridine-induced seizures in the rat brain slice, the tracing showing ictal events before, during and after application of MO. **(B)** sustained repetitive firing in current clamped neurons, tracing showing the pronounced effects of MO on sustained repetitive firing before, during and after its application.

Recording from visual cortex after 45 min perfusion of the brain slice with 4-aminopyridine (4-AP; 100 µM) revealed evoked and spontaneous ictal events occurring at regular intervals. The spontaneous ictal-like discharges were characterized by preceding negative going potentials, followed by initial tonic phases of high frequency and low amplitude and late clonic phases of high amplitude and low frequency firing with small inter-ictal events observed during refractory periods. Spontaneous ictal events were reversibly blocked by 0.1 mg/ml MO, suggesting anticonvulsant potentials of the essential oil. MO reversibly blocked the sustained repetitive firing of current clamped neuronal cells, suggesting voltage sodium channel blockade mode of action (Figure 1).

### *In vivo* Anticonvulsant Studies

#### Acute Seizure Studies

**FIGURE 2:**
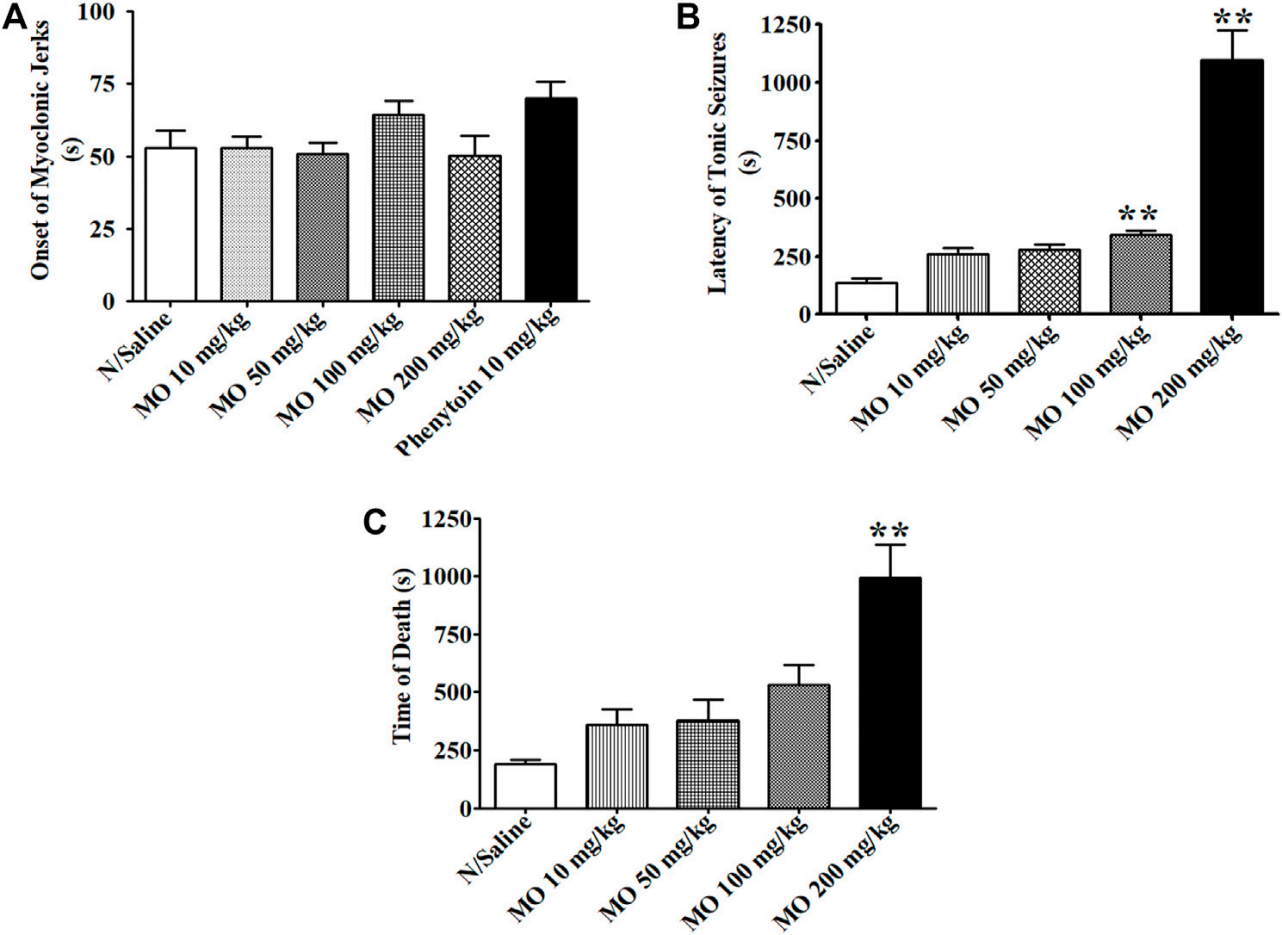
Effects of *Melissa officinalis* L. essential oil (MO) on pentylenetetrazole (PTZ, 85 mg/kg)-induced seizures in mice. **(A)** Latency of myoclonic jerks; **(B)** latency of tonic seizures; **(C)** Time of death from PTZ seizures. **Significantly different from control (*p* < 0.01, *n* = 8). Mean ± SEM.

In acute *in vivo* model, MO at doses between 10 and 100 mg/kg did not protect the mice from PTZ (85 mg/kg)-induced tonic hind limb extension. MO (200 mg/kg) and phenytoin (10 mg/kg) provided 50 and 100% protection from PTZ-induced tonic seizures and mortality in mice, respectively. MO (100 and 200 mg/kg) significantly [(F (4, 26) = 67.04, *p* < 0.01)] prolong the latency of tonic seizures in unprotected mice. Phenytoin (10 mg/kg) and MO (10, 50, 100, and 200 mg/kg) had no significant [(F (5, 42) = 2.345, *p* = 0.0603)] effect on the onset of myoclonic jerks (Figure 2). MO (100 and 200 mg/kg) protected (25 and 50%) mice against maximal electroshock seizures, respectively; phenytoin (25 mg/kg) protected 87.5% of mice from maximal electroshock seizures. MO (50, 100, and 200 mg/kg) protected 50, 75, 62.5% of mice from mortality respectively, while phenytoin (25 mg/kg) provided 87.5% protection against mortality in mice (Table 2).

**TABLE 2:** Effects of *Melissa officinalis* L. essential oil (MO) on maximal electroshock (MES)-induced seizures in mice.

Treatment (mg/kg)	No. Protected/No. of animal used	% Protection from tonic seizures	Protection from mortality	% Protection from mortality
N/Saline	0/8	0.00	0/8	0.00
MO (10)	0/8	0.00	0/8	0,00
MO (50)	0/8	0.00	4/8	50
MO (100)	2/8	25	6/8	75
MO (200)	4/8	50	5/8	62.5
Phenytoin (25)	7/8	87.5	7/8	87.5

Data are presented as number of animals protected against maximal electroshock (MES)-induced seizures out of eight animals per group. Protection of animals against mortality also was investigated. The MES test was performed 30 min after systemic (i.p.) administration of the essential oil. The essential oil from *Melissa officinalis* (MO) was suspended in 0.5% Tween80 in physiological saline solution, which was also used as control. All solutions were administered intraperitoneally (ip) at a volume of 10 ml/kg body weight.

#### PTZ Chronic Kindling Studies

**FIGURE 3:**
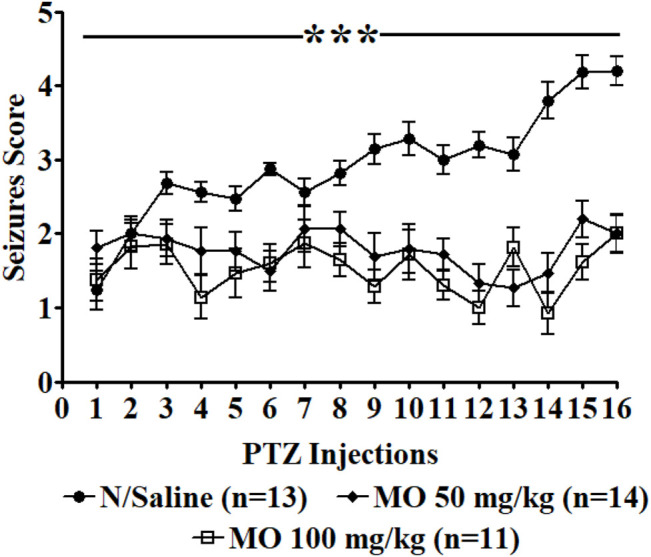
Effects of *Melissa officinalis* L. essential oil (MO) on repeated seizures induced by 35 mg/kg Pentylenetetrazole (PTZ) in rats. PTZ was administered on alternate days, 30 min after intraperitoneal MO administration. *n* = number of animals tested. Mean ± SEM. ***significantly different from control (*p* < 0.001).

**FIGURE 4:**
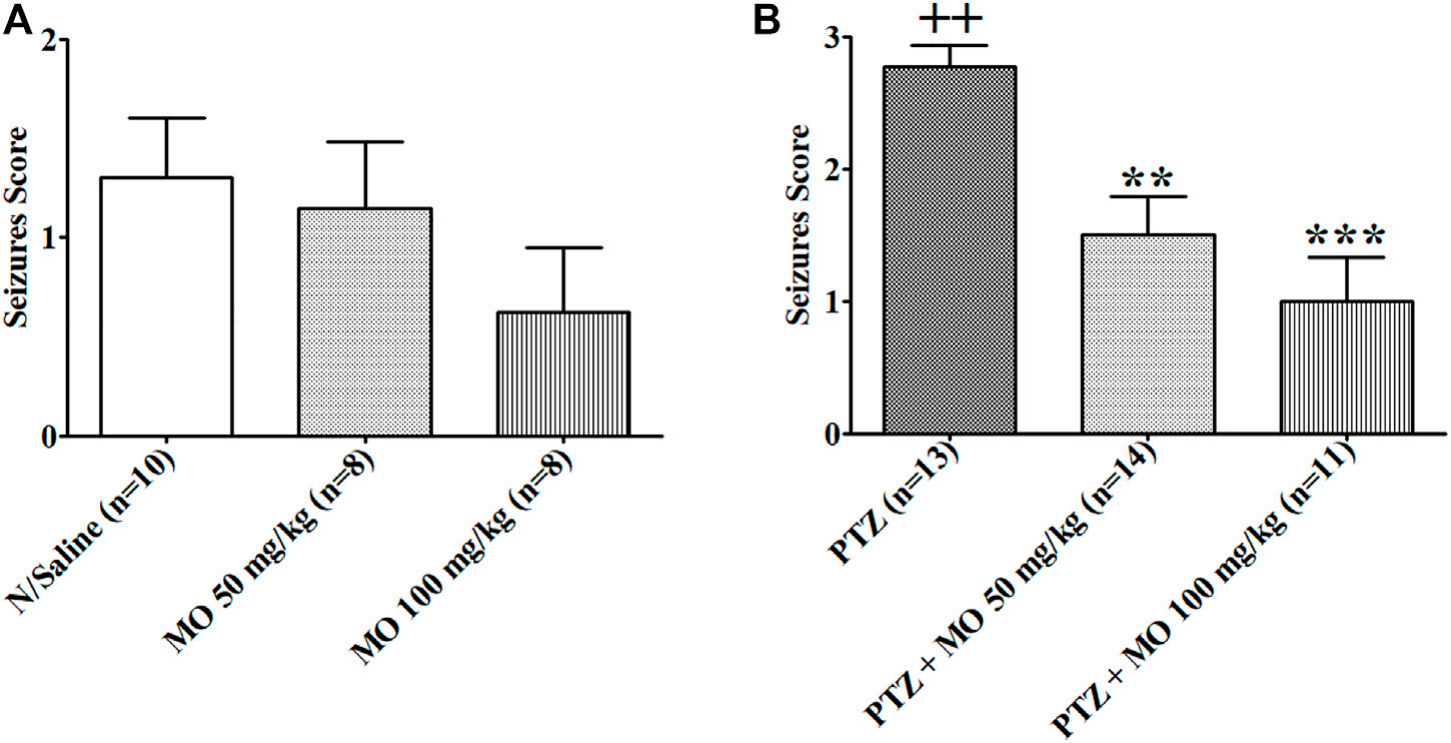
Response of rats (control vs kindled) pretreated with either saline (sal) or *Melissa officinalis* L. essential oil (MO) to a challenged dose of 32.5 mg/kg pentylenetetrazole (PTZ) injected intraperitoneally, 7 days after kindling completion. **(A)** Controlled unkindled rats **(B)** Kindled rats ++*p* < 0.01 (N/Saline vs PTZ); **p < 0.01 (PTZ vs PTZ + MO 50 mg/kg); ***p < 0.001 (PTZ vs PTZ + MO 100 mg/kg). *n* = number of animals tested. Mean ± SEM.

Repeated administration of PTZ (35 mg/kg) on alternate days led to a steady increase in convulsive activity resulting to generalized tonic–clonic seizures in vehicle treated rats, which connote an increase in brain excitability. MO (50 and 100 mg/kg, i.p.) significantly [F (2, 47) = 36.65, ***p < 0.001] reduced the seizure severity score (Figure 3). An effect on kindling and treatment [F (2, 35) = 12.28, **p < 0.01 (PTZ vs PTZ + MO 50 mg/kg); ***p < 0.001 (PTZ vs PTZ + MO 100 mg/kg)] was observed when challenged with a sub-effective dose of PTZ (32.5 mg/kg) 1 week after the last PTZ kindling injection, which suggest an enhanced brain excitability in the kindled rats. MO had no significant effects on the seizure severity in the non-kindled controlled rats during the challenge test [*F* (2, 24) = 1.243; *p* = 0.3080] (Figure 4).

### Behavioral Assessment

#### Open Field Test (OFT)

**FIGURE 5:**
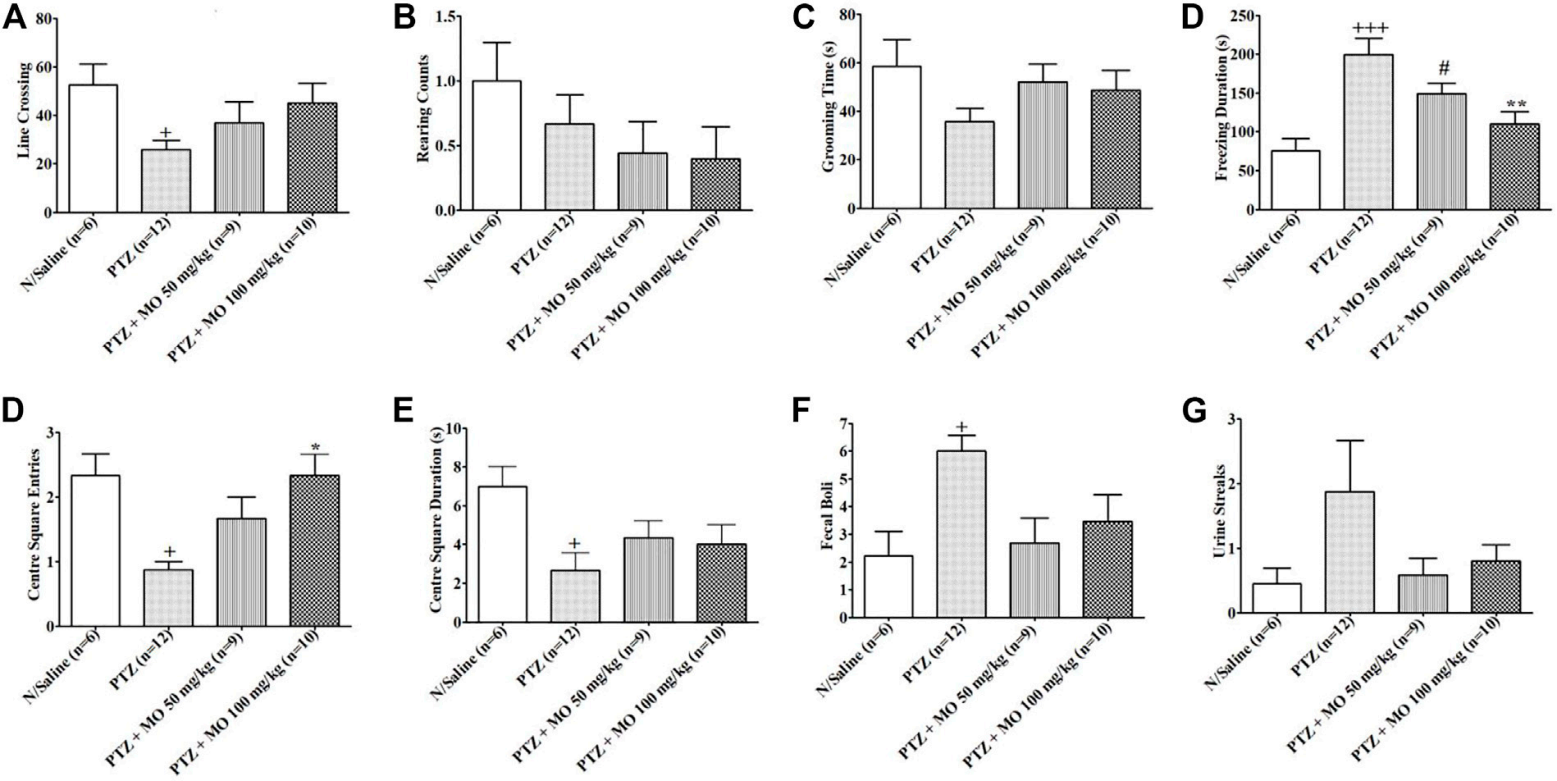
Effects of *Melissa officinalis* L. essential oil (MO) on performance in open field test in PTZ-kindled rats. **(A)** Line crossing, +*p* < 0.05 (N/Saline vs PTZ); **(B)** Rearing counts; **(C)** Grooming time; **(D)** Freezing duration, +++*p* < 0.001 (N/Saline vs PTZ), #*p* < 0.05 (N/Saline vs PTZ + MO 50 mg/kg), **p < 0.01 (PTZ vs PTZ + MO 100 mg/kg); **(E)** Centre square entries, +*p* < 0.05 (N/Saline vs PTZ), *p < 0.05 (PTZ vs PTZ + MO 100 mg/kg); **(F)** Centre square duration, +*p* < 0.05 (N/Saline vs PTZ); **(G)** Defecation, +*p* < 0.05 (N/Saline vs PTZ); **(H)** Urination. *n* = number of animals tested. Mean ± SEM.

Effects of MO on performance of PTZ-kindled rats in open field showed a significant (+*p* < 0.05) decreased in line crossing in PTZ-kindled rats (N/Saline vs PTZ). Concurrent treatment of the kindled rats with MO showed an insignificantly [F (3, 32) = 2.739, *p* = 0.0614], but dose dependently increased in line crossing activity when compared to the rats treated with PTZ only (Figure 5A). The rearing counts in kindled (PTZ treated) rats decreased when compared to saline treated rats (N/Saline vs PTZ). Treatment of the kindled rats with MO showed a further decreased in the rearing counts when compared to the animals treated with PTZ only. The results of the rearing counts (Figure 5B) were not significant [F (3, 35) = 1.015, *p* = 0.3990]. PTZ-kindled rats showed a decreased in grooming time compared to saline treated rats (N/Saline vs PTZ). Co-administration of MO and kindled rats showed an insignificantly [F (3, 33) = 1.517, *p* = 0.2301], but dose dependently increased in grooming time when compared to the PTZ treated animals (Figure 5C). The rats showed a significant increase in Freezing duration in kindled (PTZ treated) rats compared to saline treated rats {[F (3, 43) = 9.321, +++*p* < 0.001 (N/Saline vs PTZ)], which was significantly reduced with concurrent administration of MO at the dose of 100 mg/kg [F (3, 43) = 9.321, **p < 0.01 (PTZ vs PTZ + MO 100 mg/kg)]} (Figure 5D). Entries into the centre square was significantly reduced in PTZ kindled rats entries compared to saline treated rats [F (3, 12) = 7.022, +*p* < 0.05 (N/Saline vs PTZ). The centre square entries was dose dependently increased with concurrent administration of (MO) {(F (3, 12) = 7.022, *p < 0.05 (PTZ vs PTZ + MO 100 mg/kg)] (Figure 5E). Similarly, the Centre square duration was significantly (+*p* < 0.05) reduced in PTZ kindled rats compared to saline treated rats (N/Saline vs PTZ), which was dose dependently, but not significantly [(F (3, 11) = 3.708, *p* = 0.0614] increased with concurrent administration of MO (Figure 5F). Repeated administration of PTZ significantly increased the number of fecal boli [F (3, 40) = 2.992, +*p* < 0.05 (N/Saline vs PTZ)] in the kindled rats compared to the saline treated rats. The number of fecal boli insignificantly, but dose dependently reduced following concurrent administration of PTZ and MO (Figure 5G). Repeated administration of PTZ increased the number of urine streak in the kindled rats compared to the saline treated rats. The number of urine streaks were dose dependently reduced following concurrent administration of PTZ and MO with no significant effect [(F (3, 8) = 2.338, *p* = 0.0904] (Figure 5H).

#### Elevated Plus Maze (EPM)

**FIGURE 6:**
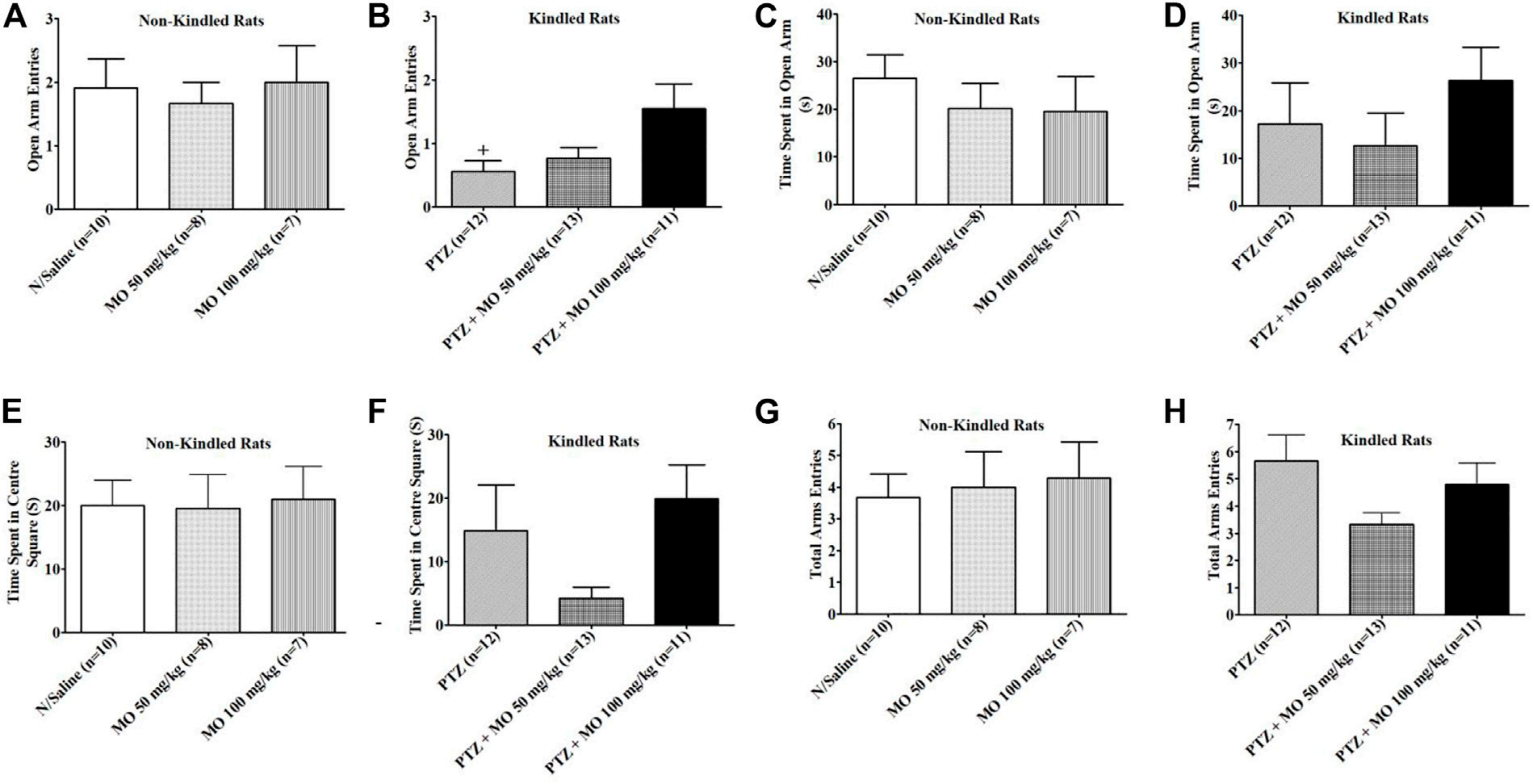
Effects of *Melissa officinalis* L. essential oil (MO) on performance of pentylenetetrazole (PTZ)-kindled rats on elevated plus maze. **(A)** Opened arm entry in non-kindled rats; **(B)** Opened arm entry in kindled rats + *p* < 0.05 (N/Saline vs PTZ); **(C)** Time spent in opened arm by non-kindled rats; **(D)** Time spent in opened arm by kindled rats; **(E)** Time spent in centre square in non-kindled rats; **(F)** Time spent in centre square in non-kindled rats; **(G)** Total arm entries by non-kindled rats; **(H)** Total arm entries by kindled rats. *n* = number of animals tested. Mean ± SEM.

Rats received 16 injections of saline, pentylenetetrazole (PTZ) or PTZ plus MO on alternate days and the effects on open arm entries, time spent in the open arm, time spent in the centre square and total arm entries by each rat were examined on an elevated plus maze (EPM). The results revealed that kindling significantly reduced open arm entries of rats [+*p* < 0.05 (N/Saline vs PTZ)]; the time spent in open arm and the centre square were also reduced, but not significant when compared to the saline treated group (N/Saline vs PTZ). The concurrent administration of PTZ and MO insignificantly increased open arm entries when compared to the PTZ treated group (Figures 6A,B). MO increased the time spent in the open arm dose dependently, but the effects were not significant at tested the doses (Figures 6C,D). MO at 50 mg/kg decreased and at 100 mg increased the time spent in the centre square compared to the PTZ treated group (Figures 6E,F). There were no significant effects on the total arm entries in both kindled and non-kindled rats (Figures 6G,H).

#### Forced Swim Test (FST)

**FIGURE 7:**
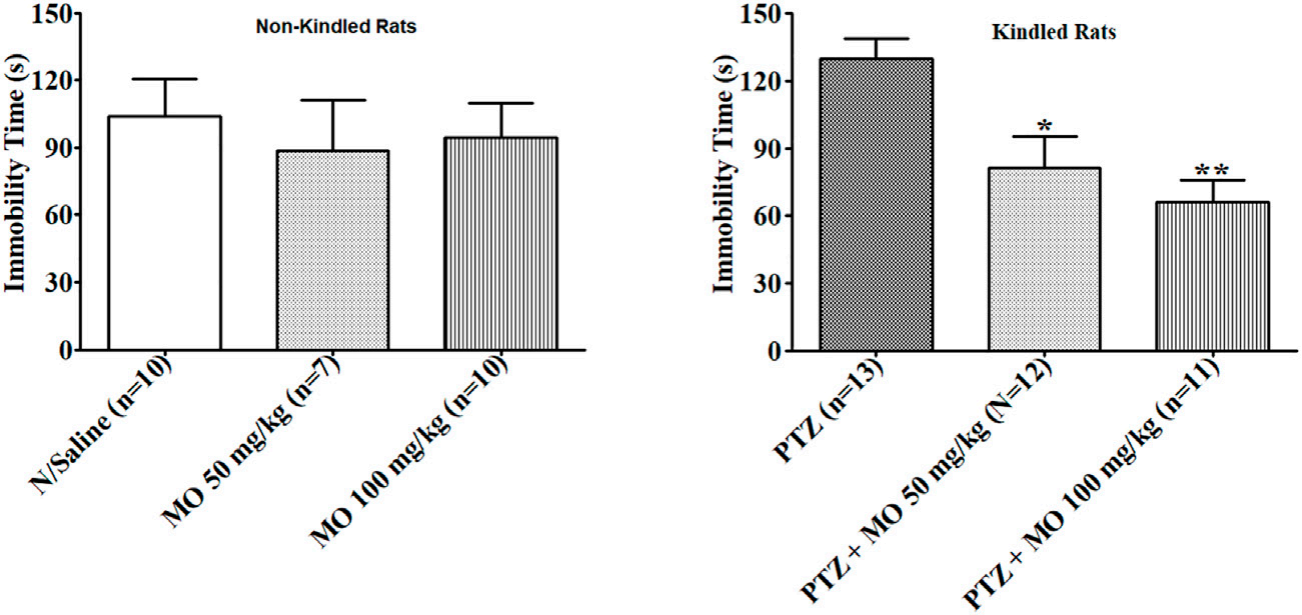
Effects of *Melissa officinalis* L. essential oil (MO) on duration of immobility in forced swim test in PTZ-kindled rats. *p < 0.05 (PTZ vs PTZ + MO 50 mg/kg); **p < 0.01 (PTZ vs PTZ + MO 100 mg/kg). *n* = number of animals tested. Mean ± SEM.

Enhanced duration of immobility was observed in rats that received repeated subconvulsive doses of PTZ in FST. The enhanced immobility time was not significant compared to saline treated rats (N/Saline vs PTZ). The effects of PTZ on duration of the immobility in the FST were significantly [F (2, 18) = 11.73, *p < 0.05 (PTZ vs PTZ + MO 50 mg/kg); **p < 0.01 (PTZ vs PTZ + MO 100 mg/kg)] reduced with concurrent administration of MO (Figure 7).

### Effects of MO on Recognition Memory of PTZ-Kindled Rats

**FIGURE 8:**
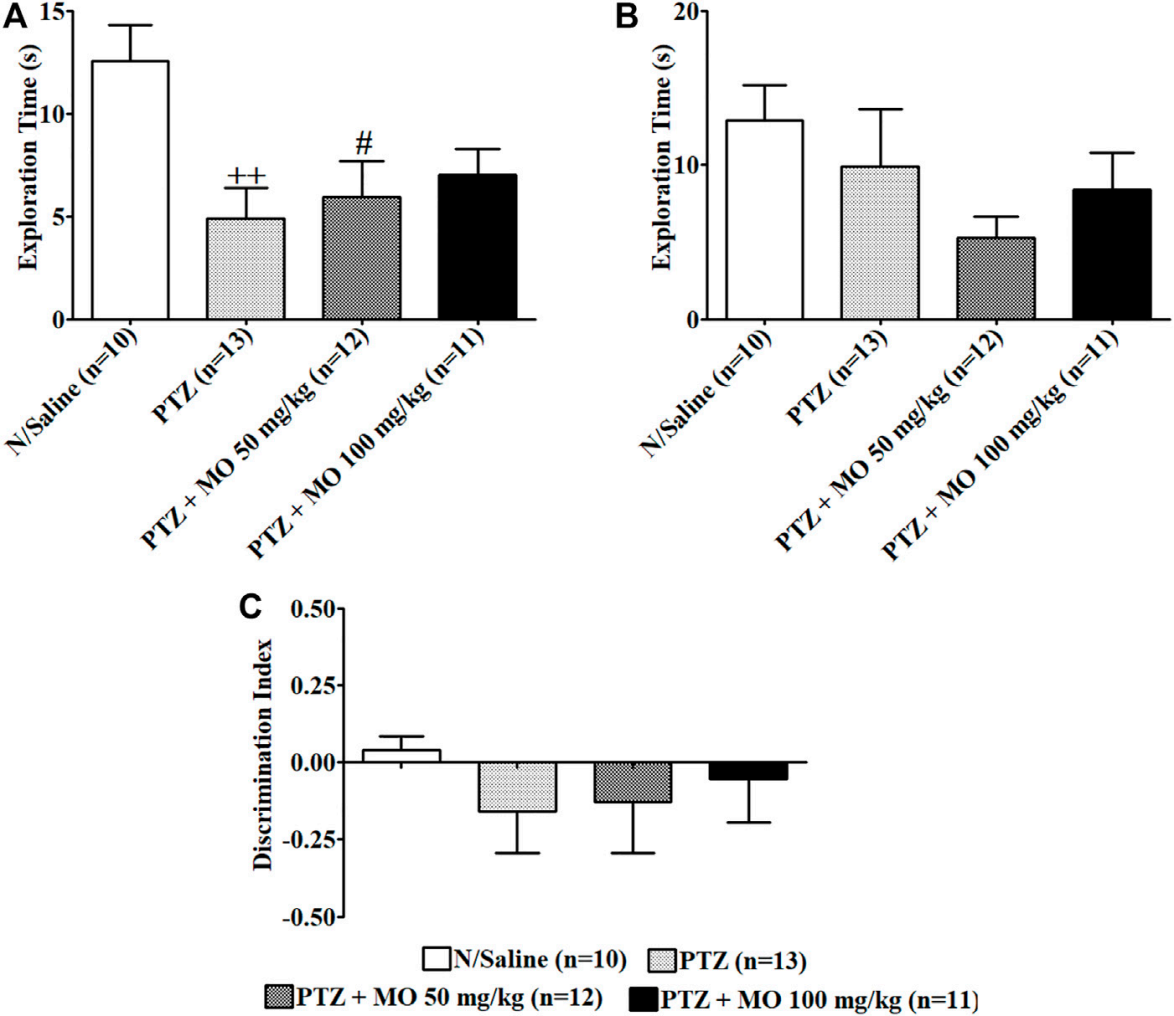
Effects of *Melissa officinalis* L. essential oil (MO) on novel object recognition test in PTZ-kindled rats. **(A)** Duration of exploration of the novel object ++*p* < 0.01 (N/Saline vs PTZ); #*p* < 0.05 (N/Saline vs PTZ + MO 50 mg/kg); **(B)** Duration of exploration of familiar object; **(C)** Discrimination Index. *n* = number of animals tested. Mean ± SEM.

Repeated administration of PTZ significantly [(F (3, 37) = 4.301, ++*p* < 0.01 (N/Saline vs PTZ)] decreased exploration of the novel object (N/Saline vs PTZ) (Figure 8A). Treatment with MO dose dependently, but not significantly increased exploration of the novel object. Similarly, repeated administration of PTZ resulted to an insignificant [(F (3, 43) = 1.546, *p* = 0.2176] reduction on exploration of familiar object (N/Saline vs PTZ), which was not significantly reduced following the administration of MO (Figure 8B). The discrimination index was insignificantly [F (3, 46) = 0.4421, *p* = 0.7241] reduced in the PTZ-alone-treated group (N/Saline vs PTZ). Pretreatment of rats with the MO insignificantly [F (3, 46) = 0.4421, *p* = 0.7241] increased the discrimination index (PTZ vs MO 50 and 100 mg/kg) (Figure 8C).

### Biochemical Assay

**FIGURE 9:**
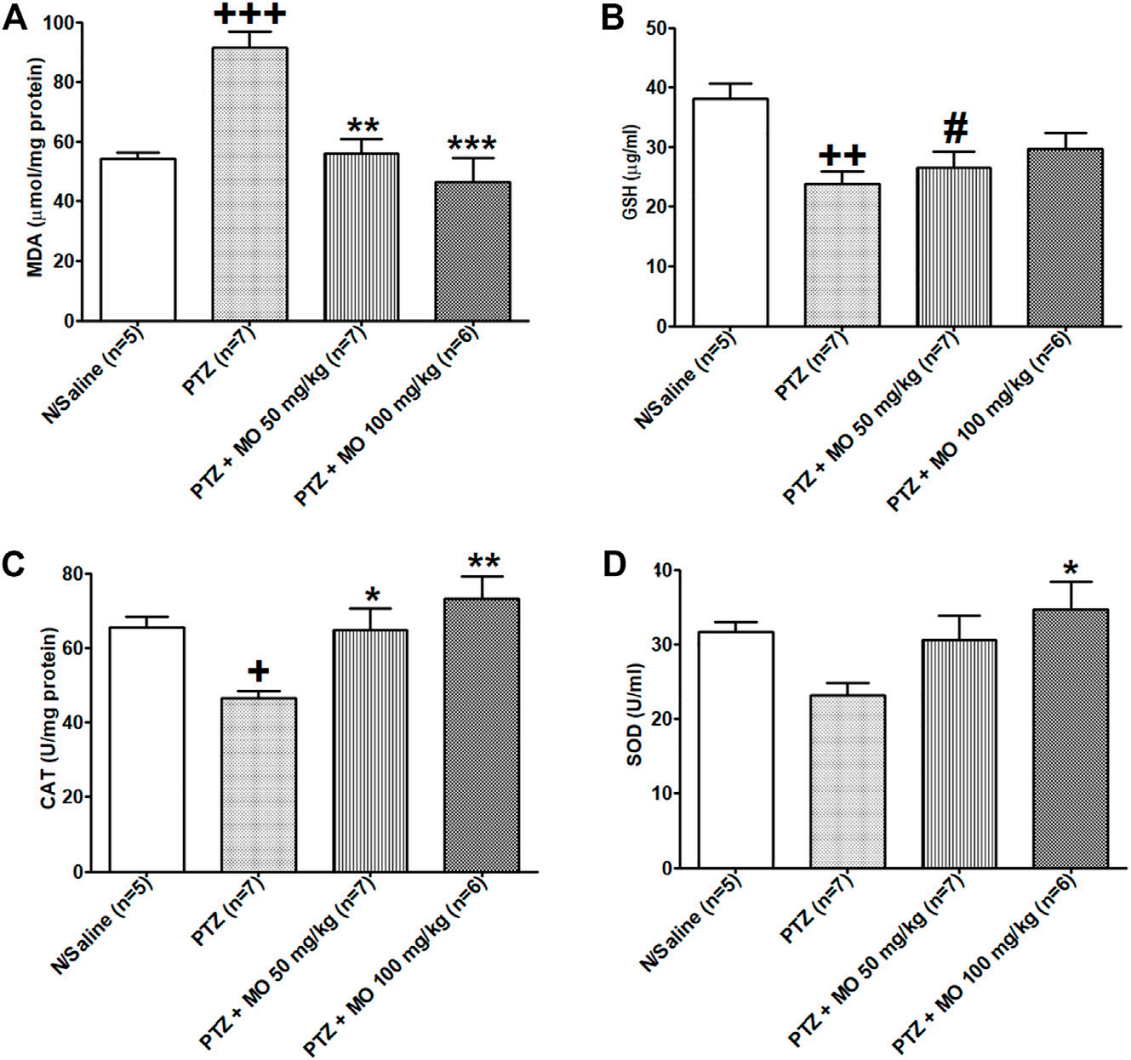
Effects of *Melissa officinalis* L. essential oil (MO) on oxidative stress markers in brains of PTZ-kindled rats. **(A)** Malondiadéhyde (MDA) level, +++*p* < 0.001 (N/Saline vs PTZ), **p < 0.01 (PTZ vs PTZ + MO 50 mg/kg), ***p < 0.001 (PTZ vs PTZ + MO 100 mg/kg); **(B)** Glutathione (GSH) level, ++*p* < 0.01 (N/Saline vs PTZ), #*p* < 0.05 (PTZ + MO 50 mg/kg vs N/Saline); **(C)** Catalase activity, +*p* < 0.05 (N/Saline vs PTZ), *p < 0.05 (PTZ vs PTZ + MO 50 mg/kg), **p < 0.01 (PTZ vs PTZ + MO 100 mg/kg); **(D)** Superoxide dismutase (SOD) activity, *p < 0.05 (PTZ vs PTZ + MO 100 mg/kg). *n* = number of animals tested. Mean ± SEM.

There was a significant [*F* (3, 17) = 12.76; +++*p* < 0.001] increase in MDA levels in kindled rats (N/Saline vs PTZ). Concurrent administration of MO with PTZ significantly [*F* (3, 17) = 12.76; **p < 0.01 (PTZ vs PTZ + MO 50 mg/kg), ***p < 0.001 (PTZ vs PTZ + MO 100 mg/kg)] reduced the MDA levels (Figure 9A). The PTZ-kindled animals showed a significant [*F* (3, 21) = 6.033; ++*p* < 0.01] decrease in GSH levels (N/Saline vs PTZ). The concurrent administration of PTZ and MO insignificantly increased the GSH levels of PTZ-kindled animals (Figure 9B). Repeated administration of PTZ-only significantly [*F* (3, 23) = 6.609; +*p* < 0.05] reduced catalase activity (N/Saline vs PTZ). Co-administration of PTZ and MO significantly [*F* (3, 23) = 6.609; *p < 0.05 (PTZ vs PTZ + MO 50 mg/kg), **p < 0.01 (PTZ vs PTZ + MO 100 mg/kg)] increased catalase activity compared to PTZ-only treated rats (Figure 9C). The PTZ treated rats showed a decrease in SOD activity that was not significant (N/Saline vs PTZ). The concurrent administration of PTZ and MO significantly [*F* (3, 24) = 3.265; *p < 0.05 (PTZ vs PTZ + MO 100 mg/kg)] increased the SOD activity (Figure 9D).

### Histopathological Studies

**FIGURE 10:**
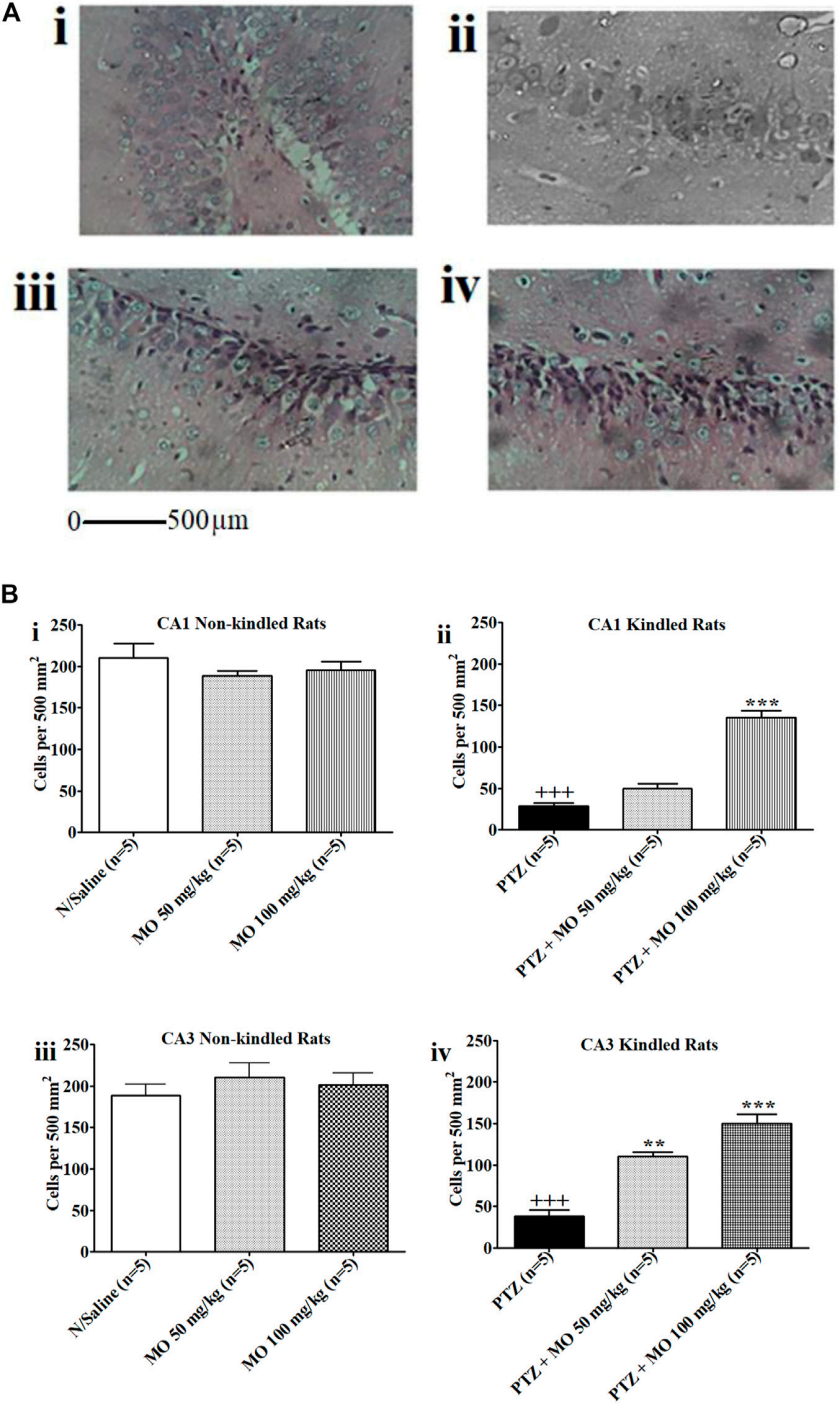
**(A)** Photomicrographs i–iv. The effects of *Melissa officinalis* L. essential oil (MO) on CA1 or CA3 neurons in the hippocampus of rats. (i) Normal Saline control showing high power view with neuronal cell nuclei and neural processes (Magnification ×400); (ii) Kindled rats treated with PTZ only (Magnification ×400); (iii) Rats treated with PTZ and 50 mg/kg of MO showing partial restoration of nuclei and neural fibrillary processes (Magnification ×400); (iv) Rats treated with PTZ and 100 mg/kg MO showing near total restoration of nuclei with their fibrillary neural processes (Magnification ×400); **(B)** Effects of *Melissa officinalis* L. essential oil (MO) on number of neurons in CA1 and CA3 in the hippocampus of rats In control animals (sal) in comparison to pentylenetetrazole-kindled mice pretreated with saline (sal) or MO. (i and ii) CA1 Non-kindled and CA1 Kindled, +++*p* < 0.001 (N/Saline vs PTZ), ***p < 0.001 (PTZ vs PTZ + MO 100 mg/kg); (iii and iv) CA3 Non-kindled and CA1 Kindled, +++*p* < 0.001 (N/Saline vs PTZ), **p < 0.01 (PTZ vs PTZ + MO 50 mg/kg), ***p < 0.001 (PTZ vs PTZ + MO 100 mg/kg). *n* = number of animals tested. Mean ± SEM.

Kindled rats treated with PTZ only showed severe degeneration of hippocampal neurons in the CA1 and CA3 regions of the rat hippocampus. Rats treated with PTZ and 50 mg/kg of MO showed partial restoration of nuclei and neural fibrillary processes, while rats treated with PTZ and 100 mg/kg MO demonstrated near total restoration of nuclei with their fibrillary neural processes (Figure 10A, i–iv). Figure 10B are histograms showing the effects of MO on the CA1 or CA3 neurons in the rat hippocampus. MO application prior to each kindling significantly counteracted neuronal cell loss in both CA1 and CA3 regions of the hippocampus. In CA1, the kindled animals showed a significant [+++*p* < 0.001; (N/Saline vs PTZ)] decrease in neuronal cells count (N/Saline vs PTZ). Concurrent administration of MO with PTZ significantly [*F* (2, 8) = 74.63; ***p < 0.001 (PTZ vs PTZ + MO 100 mg/kg)] increased the neuronal cells counts. In the CA3, PTZ-only kindled animals showed a significant [+++*p* < 0.001 (N/Saline vs PTZ)] decreased in neuronal cell counts levels (N/Saline vs PTZ). The concurrent administration of PTZ and MO significantly [*F* (2, 8) = 43.76; **p < 0.01 (PTZ vs PTZ + MO 50 mg/kg), ***p < 0.001 (PTZ vs PTZ + MO 100 mg/kg)] increased the neuronal cell counts (Figure 10B, i–iv).

## Discussion

The data presented here revealed that essential oil from *Melissa officinalis* (MO) contains neuroactive substances that have potential value in the management of epilepsy. The chemical profiles of essential oils can vary considerably within the same plant species including *M. officinalis* due to various factors (Elliott et al., 2007; Okello and Howes, 2018), it is, therefore, pertinent to chemically characterize MO such that study outcomes can be directly correlated with defined chemical compositions. The principal compounds detected in the MO were monoterpenes and sesquiterpenes, which lack nitrogen in their chemical structures, making them chemically distinct from anticonvulsant pharmaceuticals such as phenytoin, lamotrigine and carbamazepine, which contain cyclic nitrogen in their chemical structures. Here, we note this contrast is reminiscent of the discovery of the first terpenoids from sage (*Salvia* species) to inhibit acetylcholinesterase (AChE) (Perry et al., 2000; Ren et al., 2004), a target enzyme in AD, after previously known AChE inhibitors were nitrogen-containing (including plant alkaloids). Indeed, sage [*Salvia officinalis* subsp. *lavandulifolia* (Vahl) Gams] oil has shown to be beneficial in cognitive dysfunction in humans AD patients (Howes and Houghton, 2012), thus providing proof of concept that plant essential oils that are pharmacologically active *in vitro* and *in vivo*, may translate to clinical efficacy in some circumstances. This present preclinical study on MO provides a scientific basis for the potential applications of chemically-characterized oils from this species, and underpins future studies to explore the most appropriate routes of administration, considering that certain plant oil constituents, including citral (the mixture of geranial and neral, major constituents detected in the MO in this study) can mediate pharmacological effects via different routes of administration (Okello and Howes, 2018).

Previous studies have associated certain essential oil constituents with anticonvulsant activity in animal models of epilepsy, including α-pinene, linalool oxides (both isomers) and limonene (Bahr et al., 2019), which were only detected at <0.01% in the MO, so are unlikely to have significantly contributed to the anticonvulsant effects observed in the present study. However (*E*)-caryophyllene and linalool were detected at 11.56 and 1.05%, respectively, in the MO, and since these terpenoids have previously shown anticonvulsant activity *in vivo* (Bahr et al., 2019), they may have contributed to the anticonvulsant activity of the MO. Citral (a mixture of the isomers geranial and neral; detected in the MO at 25.57 and 19.54%, respectively) may also have contributed to the anticonvulsant activity of the MO in this study, as other plant oils that primarily contain this isomeric mixture of monoterpenes, are reported to be anticonvulsant *in vivo*, an action suggested to be due to modulation of γ-Aminobutyric acid (GABA) neurotransmission (Bahr et al., 2019).

In the *in vitro* studies, we induced spontaneous ictal-like epileptiform activity with 4-AP in brain slices and characterized the effects of MO (Lees et al., 2006). MO reversibly blocked spontaneous ictal events induced by 4-AP and evoked inter-ictal events during refractory periods (Lees et al., 2006).

The 4-AP enhances neuronal activity and facilitates synaptic transmission by inhibition of action potential repolarization at the presynaptic terminals of excitatory and inhibitory neurons (Thesleff, 1980; Storm, 1987), which gives rise to an increase in synaptic network interactions and entrainment of network patterns that may sustain epileptiform discharges (Avoli et al., 2002; Uva et al., 2013). The anticonvulsant effects demonstrated in this study by the oil against 4-AP-induced epileptiform seizures might be possibly via activation of potassium ion currents, the 4-AP, being a potassium channel blocker. The 4-AP mimics the electroencephalographic activity recorded in patients affected by partial epilepsy, thus effects of MO on *in vitro* brain slice 4-AP model of epilepsy are akin to anticonvulsant efficacy against partial epilepsy in humans, given that (Gonzalez-Sulser et al., 2011). MO profoundly and reversibly blocked sustained repetitive firing in current clamped neurons like lamotrigine, phenytoin, carbamazepine, lacosamide and saponins from *Ficus platyphylla* Delile (Lampl, et al., 1998; Ragsdale and Avoli, 1998; Errington et al., 2006; Chindo et al., 2009), suggesting that MO promotes fast inactivation of the voltage-gated sodium channels (Catterall, 1999; Errington et al., 2008).

In acute *in vivo* studies, MO at doses between 10 and 100 mg/kg did not protected mice from PTZ-induced tonic hind limb extension. However, MO (200 mg/kg) and phenytoin (10 mg/kg) protected 50 and 100% of the mice from PTZ-induced tonic hind limb extension and mortality, respectively. MO (100 and 200 mg/kg) significantly prolonged the latency of tonic seizures in non-protected mice. The effects of MO on PTZ-induced seizures observed in the acute studies are consistent with the findings reported by Ghayour et al. (2012) on the hydroalcoholic extract of *Melissa officinalis* (lemon balm) leaf. MO and phenytoin had no significant effect on the onset of myoclonic jerks. MO (100 and 200 mg/kg) protected 25 and 50% of mice against maximal electroshock seizures, respectively; phenytoin (25 mg/kg) protected 87.5% of mice from maximal electroshock seizures. MO (50, 100 and 200 mg/kg) protected 50, 75, and 62.5% of mice from mortality respectively, while phenytoin (25 mg/kg) protected 87.5% of mice against mortality from maximal electroshock seizures.

PTZ blocks the major inhibitory pathways mediated by GABA to induce seizure activities (Scholfield, 1982) and most conventional antiepileptic drugs augment GABA-mediated inhibition in the brain as their mode of actions (Rogawaski and Porter, 1990; MacDonald and Kelly, 1995). Studies have also implicated T-type Ca^2+^ channels and glutamatergic mechanisms in PTZ-induced convulsions. For instance, ethosuximide and felbamate that block T-type Ca^2+^ currents (Coulter et al., 1989; Meldrum, 1996) and glutamatergic excitation mediated by N-methyl-d-aspartic acid (NMDA) receptor (Nevins and Arnolde, 1989; Velisek et al., 1997), respectively, have established anticonvulsant activity against PTZ-induced seizures (MacDonald and Kelly, 1995; White, 1997). It is, therefore, probable that the anticonvulsant effects of MO against PTZ-induced seizures might as a result of enhancement of GABA-mediated inhibition, blockade of glutamatergic neurotransmission mediated by the NMDA receptor or inhibition of T-type Ca^2+^ currents. The maximal electroshock test, a simple, reliable and most widely utilized animal model of epilepsy (Sanjana et al., 2012) has a high predictive value for detecting clinically effective antiepileptic. Drugs such as phenytoin, valproate and lamotrigine, that inhibit voltage-dependent Na^+^ channels or felbamate that block glutamatergic neurotransmission mediated by the NMDA receptor (Silambujanaki et al., 2010), are known to prevent MES-induced tonic seizures. In this study, MO demonstrated a significant anticonvulsant activity in the MES model, which could possibly be via inhibition of either Na^+^ channels and/or glutamatergic neurotransmission mediated by NMDA receptors. However, previous radioligand binding studies on MO suggested little or no effect on NMDA, alpha-amino-3-hydroxy-5-methyl-4-isoxazolepropianate (AMPA) or nicotinic acetylcholine ionotropic receptors (Abuhamdah et al., 2008). Furthermore, MO may have potential value in the management of generalized tonic-clonic seizures and partial seizures, given that the oil displayed activity in the MES model (Duraisami et al., 2009; Chindo et al., 2014).

Repeated administration of a sub-convulsant dose of PTZ gradually increase convulsive activity, reflecting an increase in brain excitability that ultimately results to generalized tonic-clonic seizures. Concurrent administration of PTZ and MO during kindling and challenged tests, significantly reduced the seizure severity score in the kindled rats with no significant effects observed in the non-kindled controlled rats during the challenge experiment.

Kindling is characterized by progressive intensification of electroencephalographic and behavioral seizures evoked by subeffective doses of chemical or ramps of electrical stimuli (Chindo et al., 2015). PTZ kindling gives clue into the neurobiology of epilepsy that may be utilized to examine efficacies of antiepileptic drugs (Choudhary et al., 2013). PTZ kindling contributes to increased seizure susceptibility by altering the glutamate-mediated excitation and/or GABA-mediated inhibition (Samokhina, and Samokhin, 2018). PTZ acts on benzodiazepine (BZ) recognition sites of the GABA_A_ receptor (Ramanjaneyulu and Ticku, 1984; Rocha et al., 1996) to reduce GABA-mediated inhibition (Bradford, 1995; Rocha et al., 1996) and elevates the level of glutamate in the brain (Schünzel et al., 1992). Repeated administration of PTZ increases the number of AMPA and NMDA binding sites as well as a decrease in kainate receptor densities. MO ameliorated the seizure severity in the kindled rats via action on GABA_A_ receptor channel complex, inhibition of voltage-gated ion channel (VGIC) or inhibition of glutamate release.

Epilepsy is associated with a variety of prevalence neurological and psychiatric comorbidities including neuronal cell loss, cognitive impairments, anxiety and depression (Hermann et al., 2008; Keezer et al., 2016). Kindling is an experimental model that is widely used to assess the neurological and psychiatric comorbidities of epilepsy (Gupta et al., 2003; Mehla et al., 2010; Mishra and Goel, 2012) to provide insights into more rational therapeutic strategies in epileptic seizures. Here, we studied the effects of MO on anxiety, depression and cognitive dysfunction in PTZ-kindled rats.

Effects of MO on locomotor activity and fear-avoidance-related behaviors were studied on OFT and EPM tests (Prut and Belzung, 2003). PTZ-kindling induced a marked impairment in fear-avoidance-related behaviors and locomotor activity including reduction in line crossings, rearing counts, grooming time, entries and time spent in the centre square; and increase in freezing duration, defecation and urination, suggesting fear-avoidance-like behaviors in the kindled rats (Bindra and Thompson, 1953; Lister, 1990; Brown et al., 1999), which were prevented by concurrent administration of PTZ and MO, suggesting amelioration of fear-avoidance behaviors in the kindled rats (Ngo Bum et al., 2009; Moto et al., 2018). The open arm entries, time spent in the open arm and time spent in the centre square in the EPM test, were decreased in PTZ kindled rats, which connotes a state of fear-avoidance (Ngo Bum et al., 2009; Ennaceur and Chazot, 2016). MO reduced these parameters of fear-avoidance in PTZ kindled rats (Pitchaiah et al., 2008; Moto et al., 2013; 2018).

Depression is the most common psychiatric comorbidity observed in epileptic patients (Bragatti et al., 2011; 2014) that is generally examined in rats using tail suspension or FST (Steru et al., 1985; Singh et al., 2013). PTZ kindled rats in this study demonstrated depressive behaviors indicated by increased immobility period in forced swim test. MO ameliorated the depressive-like behavior by decreasing the immobility time in the PTZ-kindled rats.

In the NORT, repeated administration of PTZ resulted in reduction of the discriminatory index and explorations of both novel and familiar objects in the PTZ-kindled rats. Co-administration of PTZ and MO on alternate days increased the discriminatory index and explorations of the novel object and reduced the exploration of the familiar object, suggesting amelioration of cognitive deficits by MO in the PTZ-induced kindled rats (Ennaceur and Delacour, 1988; Kouémou et al., 2017).

Oxidative and nitrosative stresses, which stimulate numerous pathways that result in increased production of oxygen radicals and disruption of neuronal functions and integrity (Kovcs et al., 1996), may be associated with the generation and spreading of seizure activity (Chang and Yu, 2010). Mitochondrial dysfunction and oxidative stress are mplicated the pathophysiology of epilepsy (Kumar et al., 2014) and free radicals are found to be consistently elevated in epilepsy (Aguiar et al., 2013). PTZ-kindling conduces reactive oxygen species (ROS) production (Rauca et al., 2004; Patsoukis et al., 2005) and ROS production agrees with decrease in antioxidant enzymes (Devi et al., 2008). In this study, MDA levels significantly increased while GSH concentrations, and CAT and SOD activities were reduced in the PTZ-kindled rats, suggesting that chronic administration of subeffective doses of PTZ promotes oxidative stress (Taiwe et al., 2015). MO ameliorated oxidative stress by increasing GSH levels, SOD and CAT activities; and decreasing MDA levels in the PTZ-kindled rats, thus corroborating the therapeutic benefits of MO in the management of epilepsy.

Histological examinations of brains from the PTZ-kindled rats provides insights into decline in number and density of hippocampal neurons. Energy deprivation coupled with calcium-dependent modulations of NMDA currents (Grishin et al., 2004) (Gee et al., 2006) are implicated in neuronal cell loss after repetitive seizure activity. PTZ kindling induces unilateral low blood perfusion resulting to a corresponding decline in glucose metabolism and loss of hippocampal neurons (Samokhina and Samokhin, 2018). MO pretreatment ameliorated neuronal cell loss in hippocampus of the kindled rats by undetermined mechanism(s).

## Conclusion

Our results revealed that MO contains biologically active chemical constituents with potential anticonvulsant properties. Neuroactive principles in MO reversibly blocked both spontaneous ictal like discharges in brain slice 4-AP model of epilepsy and sustained repetitive firing in current clamped neurons, suggesting anticonvulsant effects and voltage-gated sodium channel blockade mode of action, respectively. MO protected mice from PTZ- and MES-induced seizures and mortalities; and ameliorated seizure severity, fear-avoidance, depressive-like behavior, oxidative stress and neuronal cells loss in PTZ-kindled rats, thus, corroborating the potential therapeutic benefits of MO in the management of epilepsy. These findings merit further evaluation of MO and its constituents for potential use as an adjunctive therapy for epileptic patients to prevent the emergence of major neuropsychiatric symptoms associated with the disease.

## Data Availability

The original contributions presented in the study are included in the article/Supplementary Material, further inquiries can be directed to the corresponding author.

## Supplementary Material

Supplementary Figure S1 GC-MS total ion chromatogram of the *Melissa officinalis* essential oil, with chromatography performed on a 30 m × 0.25 mm ID × 0.25 µm DB-5MS column.

## Abbreviations

4-AP, 4-aminopyridine; AD, Alzheimer’s disease; AEDs, Antiepileptic drugs; AMPA, alpha-amino-3-hydroxy-5-methyl-4-isoxazolepropianate; BZ, Benzodiazepine; CAT, Catalase; CBZ, Carbamazepine; CM, Cerebral malaria; CNS, Central nervous system; EPM, Elevated plus Maze; FST, Forced swim test; GABA, γ‐aminobutyric acid; GC-MS, Gas Chromatography-Mass Spectrometry; GSH, Glutathione; MDA Malondialdehyde; MES, Maximal electroshock seizures; MO, *Melissa officinalis* essential oil; NMDA, N-methyl-D-aspartate; NORT, Novel object recognition test; PTZ, Pentylenetetrazole; SOD Superoxide dismutase; SRF, Sustained repetitive firing; VGSCs, Voltage-gated sodium channels.

## References

[B1] Abuhamdah S. Huang L. Elliott M. S. Howes M. J. Ballard C. Holmes C. (2008). Pharmacological Profile of an Essential Oil Derived from *Melissa officinalis* with Anti-agitation Properties: Focus on Ligand-Gated Channels. J. Pharm. Pharmacol. 60, 377–384. 10.1211/jpp.60.3.0014 18284819

[B2] Adams R. P. (2007). Identification of Essential Oil Components by Gas Chromatography/Mass Spectroscopy. Illinois, USA: Allured Publishing Corporation.

[B3] Aguiar C. C. Almeida A. B. Araújo P. V. Vasconcelos G. S. Chaves E. M. do Vale O. C. (2013). Effects of Agomelatine on Oxidative Stress in the Brain of Mice after Chemically Induced Seizures. Cell. Mol. Neurobiol. 33, 825–835. 10.1007/s10571-013-9949-0 23801192 PMC11498010

[B4] Annegers J. F. Hauser W. A. Beghi E. Nicolosi A. Kurland L. T. (1988). The Risk of Unprovoked Seizures after Encephalitis and Meningitis. Neurology 38, 1407–1410. 10.1212/wnl.38.9.1407 3412588

[B5] Avoli M. D'Antuono M. Louvel J. Köhling R. Biagini G. Pumain R. (2002). Network and Pharmacological Mechanisms Leading to Epileptiform Synchronization in the Limbic System *In Vitro* . Prog. Neurobiol. 68, 167–207. 10.1016/s0301-0082(02)00077-1 12450487

[B6] Bahr T. A. Rodriguez D. Beaumont C. Allred K. (2019). The Effects of Various Essential Oils on Epilepsy and Acute Seizure: A Systematic Review. Evid. Based Complement. Altern. Med. 2019, 1–14. 10.1155/2019/6216745 PMC6556313 31239862

[B7] Becker A. Grecksch G. Brosz M. (1995). Antiepileptic Drugs-Ttheir Effects on Kindled Seizures and Kindling-Induced Learning Impairments. Pharmacol. Biochem. Behav. 52, 453–459. 10.1016/0091-3057(95)00137-l 8545459

[B8] Bedford H. de Louvois J. Halket S. Peckham C. Hurley R. Harvey D. (2001). Meningitis in Infancy in England and Wales: Follow up at Age 5 Years. BMJ 323, 533–536. 10.1136/bmj.323.7312.533 11546697 PMC48156

[B9] Benneh C. K. Biney R. P. Tandoh A. Ampadu F. A. Adongo D. W. Jato J. (2018). *Maerua angolensis* DC. (Capparaceae) Stem Bark Extract Protects against Pentylenetetrazole-Induced Oxidative Stress and Seizures in Rats. Evid. Based Complement. Alternat Med. 2018, 9684138. 10.1155/2018/9684138 29853980 PMC5954932

[B10] Bhat J. U. Nizami Q. Aslam M. Asiaf A. Ahmad S. T. Parray S. A. (2012). Antiepileptic Activity of the Whole Plant Extract of *Melissa officinalis* in Swiss Albino Mice. Int. J. Pharm. Sc. Res. 3, 886–889. 10.13040/IJPSR.0975-8232.3(3).886-89

[B11] Bindra D. Thompson W. R. (1953). An Evaluation of Defecation and Urination as Measures of Fearfulness. J. Comp. Physiol. Psychol. 46, 43–45. 10.1037/h0057952 13034953

[B12] Blumenthal M. Goldberg A. Brinckmann J. (2000). Herbal Medicine Expanded Commission E Mongraphs. Integr. Med. Com 123, 230–232.

[B13] Bradford H. F. (1995). Glutamate, GABA and Epilepsy. Prog. Neurobiol. 47, 477–511. 10.1016/0301-0082(95)00030-5 8787032

[B14] Bragatti J. A. Bandeira I. C. de Carvalho A. M. Abujamra A. L. Leistner-Segal S. Bianchin M. M. (2014). Tryptophan Hydroxylase 2 (TPH2) Gene Polymorphisms and Psychiatric Comorbidities in Temporal Lobe Epilepsy. Epilepsy Behav. 32, 59–63. 10.1016/j.yebeh.2014.01.007 24491795

[B15] Bragatti J. A. Torres C. M. Londero R. G. Martin K. C. Souza A. C. Hidalgo M. P. (2011). Prevalence of Psychiatric Comorbidities in Temporal Lobe Epilepsy in a Southern Brazilian Population. Arq. Neuropsiquiatr. 69, 159–165. 10.1590/s0004-282x2011000200003 21537552

[B16] Brown R. E. Corey S. C. Moore A. K. (1999). Differences in Measures of Exploration and Fear in MHC-Congenic C57BL/6J and B6-H-2k Mice. Behav. Genet. 29, 263–271. 10.1023/a:1021694307672

[B17] Bum E. N. Schmutz M. Meyer C. Rakotonirina A. Bopelet M. Portet C. (2001). Anticonvulsant Properties of the Methanolic Extract of *Cyperus articulatus* (Cyperaceae). J. Ethnopharmacol. 76, 145–150. 10.1016/s0378-8741(01)00192-1 11390127

[B18] Catterall W. A. (1999). Molecular Properties of Brain Sodium Channels: an Important Target for Anticonvulsant Drugs. Adv. Neurol. 79, 441–456. 10514834

[B19] Chang S. J. Yu B. C. (2010). Mitochondrial Matters of the Brain: Mitochondrial Dysfunction and Oxidative Status in Epilepsy. J. Bioenerg. Biomembr. 42, 457–459. 10.1007/s10863-010-9317-4 21086030

[B20] Chindo B. A. Adzu B. Yahaya T. A. Gamaniel K. S. (2012). Ketamine-enhanced Immobility in Forced Swim Test: a Possible Animal Model for the Negative Symptoms of Schizophrenia. Prog. Neuropsychopharmacol. Biol. Psychiatry 38, 310–316. 10.1016/j.pnpbp.2012.04.018 22561603

[B21] Chindo B. A. Anuka J. A. McNeil L. Yaro A. H. Adamu S. S. Amos S. (2009). Anticonvulsant Properties of Saponins from *Ficus platyphylla* Stem Bark. Brain Res. Bull. 78, 276–282. 10.1016/j.brainresbull.2008.12.005 19111909

[B22] Chindo B. A. Schröder H. Becker A. (2015). Methanol Extract of *Ficus Platyphylla* Ameliorates Seizure Severity, Cognitive Deficit and Neuronal Cell Loss in Pentylenetetrazole-Kindled Mice. Phytomedicine 22, 86–93. 10.1016/j.phymed.2014.10.005 25636876

[B23] Chindo B. A. Ya'U J. Danjuma N. M. Okhale S. E. Gamaniel K. S. Becker A. (2014). Behavioral and Anticonvulsant Effects of the Standardized Extract of *Ficus platyphylla* Stem Bark. J. Ethnopharmacol. 154, 351–360. 10.1016/j.jep.2014.03.061 24754912

[B24] Choudhary K. M. Mishra A. Poroikov V. V. Goel R. K. (2013). Ameliorative Effect of Curcumin on Seizure Severity, Depression like Behavior, Learning and Memory Deficit in post-pentylenetetrazole-kindled Mice. Eur. J. Pharmacol. 704, 33–40. 10.1016/j.ejphar.2013.02.012 23461849

[B25] Cotella E. M. Lascano I. M. Levin G. M. Suárez M. M. (2009). Amitriptyline Treatment under Chronic Stress Conditions: Effect on Circulating Catecholamines and Anxiety in Early Maternally Separated Rats. Int. J. Neurosci. 119, 664–680. 10.1080/00207450802330611 19283592

[B26] Coulter D. A. Huguenard J. R. Prince D. A. (1989). Characterization of Ethosuximide Reduction of Low-Threshold Calcium Current in Thalamic Neurons. Ann. Neurol. 25, 582–593. 10.1002/ana.410250610 2545161

[B27] Detke M. J. Rickels M. Lucki I. (1995). Active Behaviors in the Rat Forced Swimming Test Differentially Produced by Serotonergic and Noradrenergic Antidepressants. Psychopharmacology (Berl) 121, 66–72. 10.1007/BF02245592 8539342

[B28] Devi P. U. Manocha A. Vohora D. (2008). Seizures, Antiepileptics, Antioxidants and Oxidative Stress: an Insight for Researchers. Expert Opin. Pharmacother. 9, 3169–3177. 10.1517/14656560802568230 19040338

[B29] Dubé C. M. Brewster A. L. Baram T. Z. (2009). Febrile Seizures: Mechanisms and Relationship to Epilepsy. Brain Dev. 31, 366–371. 10.1016/j.braindev.2008.11.010 19232478 PMC2698702

[B30] Elliott M. S. J. Abuhamdah S. Howes M-J. R. Lees G. Ballard C. G. Holmes C. (2007). The Essential Oils from *Melissa officinalis* L. And *Lavandula angustifolia* Mill. as Potential Treatment for Agitation in People with Severe Dementia. Int. J. Essent. Oil Ther. 1, 143–152.

[B31] Engel J. (2001). A Proposed Diagnostic Scheme for People with Epileptic Seizures and with Epilepsy: Report of the ILAE Task Force on Classification and Terminology. Epilepsia 42, 796–803. 10.1046/j.1528-1157.2001.10401.x 11422340

[B32] Ennaceur A. Chazot P. L. (2016). Preclinical Animal Anxiety Research - Flaws and Prejudices. Pharmacol. Res. Perspect. 4 (2), e00223. 10.1002/prp2.223 27069634 PMC4804324

[B33] Ennaceur A. Delacour J. (1988). A New One-Trial Test for Neurobiological Studies of Memory in Rats. 1: Behavioral Data. Behav. Brain Res. 31, 47–59. 10.1016/0166-4328(88)90157-x 3228475

[B34] Errington A. C. Coyne L. Stöhr T. Selve N. Lees G. (2006). Seeking a Mechanism of Action for the Novel Anticonvulsant Lacosamide. Neuropharmacology 50, 1016–1029. 10.1016/j.neuropharm.2006.02.002 16620882

[B35] Errington A. C. Stöhr T. Heers C. Lees G. (2008). The Investigational Anticonvulsant Lacosamide Selectively Enhances Slow Inactivation of Voltage-Gated Sodium Channels. Mol. Pharmacol. 73, 157–169. 10.1124/mol.107.039867 17940193

[B36] Errington A. C. Stöhr G. Lees G. (2005). Voltage Gated Ion Channels: Targets for Anticonvulsant Drugs. Curr. Top. Med. Chem. 5, 15–30. 10.2174/1568026053386872 15638775

[B37] Gee C. E. Benquet P. Raineteau O. Rietschin L. Kirbach S. W. Gerber U. (2006). NMDA Receptors and the Differential Ischemic Vulnerability of Hippocampal Neurons. Eur. J. Neurosci. 23, 2595–2603. 10.1111/j.1460-9568.2006.04786.x 16817862

[B38] Ghayour M. B. Behnam-Rassouli M. Ghayour N. Tehranipour M. Kamyabi-Abkooh A. (2012). Investigating the Anti-epileptic and Sedative Effects of Hydroalcoholic Extract of *Melissa officinalis* (Lemon Balm) Leaf on Pentylenetetrazole Induced Epileptiform Seizures in Wistar Rat. J. Med. Plants 11, 64–73.

[B39] Gidal B. E. Garnett W. R. Graves N. (2002). “Epilepsy,” in Pharmacotherapy: A Pathophysiologic Approach. Editors Dipiro J. T. Talbert R. L. Yee G. C. Matzke G. R. Wells B. G. Posey M. L. 5th edition (New York: McGraw Hill Co. Inc.), 1031–1059.

[B40] Gonzalez-Sulser A. Wang J. Motamedi G. K. Avoli M. Vicini S. Dzakpasu R. (2011). The 4-aminopyridine *In Vitro* Epilepsy Model Analyzed with a Perforated Multi-Electrode Array. Neuropharmacology 60 (7-8), 1142–1153. 10.1016/j.neuropharm.2010.10.007 20955719 PMC3032005

[B113] Góth L. (1991). A Simple Method for Determination of Serum Catalase Activity and Revision of Reference Range. Clin. Chim. Acta 196, 143–151. 10.1016/0009-8981(91)90067-m 2029780

[B41] Grishin A. A. Gee C. E. Gerber U. Benquet P. (2004). Differential Calcium-dependent Modulation of NMDA Currents in CA1 and CA3 Hippocampal Pyramidal Cells. J. Neurosci. 24, 350–355. 10.1523/JNEUROSCI.4933-03.2004 14724233 PMC6729976

[B42] Gupta Y. K. Veerendra Kumar M. H. Srivastava A. K. (2003). Effect of *Centella asiatica* on Pentylenetetrazole-Induced Kindling, Cognition and Oxidative Stress in Rats. Pharmacol. Biochem. Behav. 74, 579–585. 10.1016/s0091-3057(02)01044-4 12543222

[B43] Hermann B. Seidenberg M. Jones J. (2008). The Neurobehavioural Comorbidities of Epilepsy: Can a Natural History Be Developed? Lancet Neurol. 7, 151–160. 10.1016/S1474-4422(08)70018-8 18207113

[B44] Holopainen I. E. (2008). Seizures in the Developing Brain: Cellular and Molecular Mechanisms of Neuronal Damage, Neurogenesis and Cellular Reorganization. Neurochem. Int. 52, 935–947. 10.1016/j.neuint.2007.10.021 18093696

[B45] Howes M. J. Houghton P. J. (2012). Ethnobotanical Treatment Strategies against Alzheimer's Disease. Curr. Alzheimer Res. 9, 67–85. 10.2174/156720512799015046 22329652

[B46] Jain N. N. Ohal C. C. Shroff S. K. Bhutada R. H. Somani R. S. Kasture V. S. (2003). *Clitoria ternatea* and the CNS. Pharmacol. Biochem. Behav. 75, 529–536. 10.1016/s0091-3057(03)00130-8 12895670

[B47] Janina M. S. (2003). Melissa officinalis . Int. J. Aromather. 10, 132–139.

[B48] Jones N. C. Salzberg M. R. Kumar G. Couper A. Morris M. J. O'Brien T. J. (2008). Elevated Anxiety and Depressive-like Behavior in a Rat Model of Genetic Generalized Epilepsy Suggesting Common Causation. Exp. Neurol. 209, 254–260. 10.1016/j.expneurol.2007.09.026 18022621

[B49] Keezer M. R. Sisodiya S. M. Sander J. W. (2016). Comorbidities of Epilepsy: Current Concepts and Future Perspectives. Lancet Neurol. 15, 106–115. 10.1016/S1474-4422(15)00225-2 26549780

[B50] Kittler J. Krüger H. Lohwasser U. Ulrich D. Zeiger B. Schütze W. (2018b). Evaluation of 28 Balm and Lemon Balm (*Melissa officinalis*) Accessions for Content and Composition of Essential Oil and Content of Rosmarinic Acid. Genet. Resour. Crop Evol. 65, 745–757. 10.1007/s10722-017-0568-3

[B51] Kittler J. Krüger H. Ulrich D. Zeiger B. Schütze W. Böttcher C. (2018a). Content and Composition of Essential Oil and Content of Rosmarinic Acid in Lemon Balm and Balm Genotypes (*Melissa officinalis*). Genet. Resour. Crop Evol. 65, 1517–1527. 10.1007/s10722-018-0635-4

[B52] Kouémou N. E. Taiwe G. S. Moto F. C. O. Pale S. Ngoupaye G. T. Njapdounke J. S. K. (2017). Nootropic and Neuroprotective Effects of *Dichrocephala integrifolia* on Scopolamine Mouse Model of Alzheimer's Disease. Front. Pharmacol. 8 (847), 847–910. 10.3389/fphar.2017.00847 29209218 PMC5702348

[B53] Kovacs I. Papathomas T. V. Yang M. Fehér A. (1996). When the Brain Changes its Mind: Interocular Grouping during Binocular Rivalry. Proc. Natl. Acad. Sci. 93, 15508–15511. 10.1073/pnas.93.26.15508 8986842 PMC26435

[B54] Kroczka B. Branski P. Palucha A. Pilc A. Nowak G. (2001). Antidepressant-like Properties of Zinc in Rodent Forced Swim Test. Brain Res. Bull. 55, 297–300. 10.1016/s0361-9230(01)00473-7 11470330

[B55] Kulkarni P. D. Ghaisas M. M. Chivate N. D. Sankpal P. S. (2011). Memory Enhancing Activity of *Cissampelos pariera* in Mice. Int. J. Pharm. Pharm. Sci. 3, 206–211.

[B56] Kumar A. Lalitha S. Mishra J. (2014). Hesperidin Potentiates the Neuroprotective Effects of Diazepam and Gabapentin against Pentylenetetrazole-Induced Convulsions in Mice: Possible Behavioral, Biochemical and Mitochondrial Alterations. Indian J. Pharmacol. 46, 309–315. 10.4103/0253-7613.132180 24987179 PMC4071709

[B57] Lampl I. Schwindt P. Crill W. (1998). Reduction of Cortical Pyramidal Neuron Excitability by the Action of Phenytoin on Persistent Na+ Current. J. Pharmacol. Exp. Ther. 284, 228–237. 9435183

[B58] Lees G. Leach M. J. (1993). Studies on the Mechanism of Action of the Novel Anticonvulsant Lamotrigine (Lamictal) Using Primary Neurological Cultures from Rat Cortex. Brain Res. 612, 190–199. 10.1016/0006-8993(93)91660-k 7687190

[B59] Lees G. Stöhr T. Errington A. C. (2006). Stereoselective Effects of the Novel Anticonvulsant Lacosamide against 4-AP Induced Epileptiform Activity in Rat Visual Cortex *In Vitro* . Neuropharmacology 50, 98–110. 10.1016/j.neuropharm.2005.08.016 16225894

[B60] Lindholm J. S. Autio H. Vesa L. Antila H. Lindemann L. Hoener M. C. (2012). The Antidepressant-like Effects of Glutamatergic Drugs Ketamine and AMPA Receptor Potentiator LY 451646 Are Preserved in Bdnf^+^/^-^Heterozygous Null Mice. Neuropharmacology 62, 391–397. 10.1016/j.neuropharm.2011.08.015 21867718

[B61] Lister R. G. (1990). Ethologically-based Animal Models of Anxiety Disorders. Pharmacol. Ther. 46, 321–340. 10.1016/0163-7258(90)90021-s 2188266

[B62] Löscher W. (2002). Current Status and Future Directions in the Pharmacotherapy of Epilepsy. Trends Pharmacol. Sci. 23, 113–118. 10.1016/S0165-6147(00)01974-X 11879677

[B63] MacDonald R. L. Kelly K. M. (1995). Antiepileptic Drug Mechanisms of Action. Epilepsia 36, S2–S12. 10.1111/j.1528-1157.1995.tb05996.x 8784210

[B64] Meftahizade H. Moradkhani H. Naseri B. Lotfi M. Naseri A. (2010). Improved *In Vitro* Culture and Micropropagation of Different *Melissa officinalis* L. Genotypes. J. Med. Plant Res. 4, 240–246.

[B65] Mehla J. Reeta K. H. Gupta P. Gupta Y. K. (2010). Protective Effect of Curcumin against Seizures and Cognitive Impairment in a Pentylenetetrazole-Kindled Epileptic Rat Model. Life Sci. 87, 596–603. 10.1016/j.lfs.2010.09.006 20840851

[B66] Meldrum B. S. (1996). Update on the Mechanism of Action of Antiepileptic Drugs. Epilepsia 37 (Suppl. 6), S4–S11. 10.1111/j.1528-1157.1996.tb06038.x 8941036

[B67] Miranda D. D. C. Brucki S. M. D. (2014). Epilepsy in Patients with Alzheimer's Disease: A Systematic Review. Dement Neuropsychol. 8, 66–71. 10.1590/S1980-57642014DN81000010 29213881 PMC5619450

[B68] Mishra A. Goel R. K. (2012). Age Dependent Learning and Memory Deficit in Pentylenetetrazol Kindled Mice. Eur. J. Pharmacol. 674, 315–320. 10.1016/j.ejphar.2011.11.010 22119078

[B69] Misra H. P. Fridovich I. (1972). The Role of Superoxide Anion in the Autoxidation of Epinephrine and a Simple Assay for Superoxide Dismutase. J. Biol. Chem. 247, 3170–3175. 10.1016/S0021-9258(19)45228-9 4623845

[B70] Moron M. S. Depierre J. W. Mannervik B. (1979). Levels of Glutathione, Glutathione Reductase and Glutathione S-Transferase Activities in Rat Lung and Liver. Biochim. Biophys. Acta 582, 67–78. 10.1016/0304-4165(79)90289-7 760819

[B71] Moto F. C. O. Arsa'a A. Ngoupaye G. T. Taiwe G. S. Njapdounke J. S. K. Kandeda A. K. (2018). Anxiolytic and Antiepileptic Properties of the Aqueous Extract of *Cissus quadrangularis* (Vitaceae) in Mice Pilocarpine Model of Epilepsy. Front. Pharmacol. 9 (751), 751–810. 10.3389/fphar.2018.00751 30065650 PMC6056655

[B72] Moto F. C. O. Ngo Bum E. Talla E. Taiwe G. S. Ngoupaye G. T. (2013). Anxiolytic-like Effects of the Decoction of *Psorospermum febrifugum* in Mice. Asian J. Pharm. Health Sci. 3, 372–379.

[B73] Murthy J. M. Prabhakar S. (2008). Bacterial Meningitis and Epilepsy. Epilepsia 49 (Suppl. 6), 8–12. 10.1111/j.1528-1167.2008.01750.x 18754955

[B74] Nevins M. E. Arnolde S. M. (1989). A Comparison of the Anticonvulsant Effects of Competitive and Non-competitive Antagonists of the N-Methyl-D-Aspartate Receptor. Brain Res. 503, 1–4. 10.1016/0006-8993(89)91695-8 2558775

[B75] Ngo Bum E. Taiwe G. S. Moto F. C. Ngoupaye G. T. Nkantchoua G. C. Pelanken M. M. (2009). Anticonvulsant, Anxiolytic, and Sedative Properties of the Roots of *Nauclea latifolia* Smith in Mice. Epilepsy Behav. 15, 434–440. 10.1016/j.yebeh.2009.05.014 19560975

[B76] Ngoungou E. B. Preux P. M. (2008). Cerebral Malaria and Epilepsy. Epilepsia 49, 19–24. 10.1111/j.1528-1167.2008.01752.x 18754957

[B77] NIST (2008). NIST/EPA/NIH Mass Spectral Database. Gaithersburg, USA: National Institute of Standards and Technology.

[B78] Ohkawa H. Ohishi N. Yagi K. (1979). Assay for Lipid Peroxides in Animal Tissues by Thiobarbituric Acid Reaction. Anal. Biochem. 95, 351–358. 10.1016/0003-2697(79)90738-3 36810

[B79] Okello E. J. Howes M-J. (2018). “Essential Oils and aromas that Affect Mood and Cognition,” in Routledge International Handbook of Psychobiology. first edition (United Kingdom: Tayor & Francis Group). 10.4324/9781315642765-15

[B80] Pai P. Sanjana K. Deepa B. Shyamjith M. Rao S. (2012). Effect of Artesunate on Maximal Electroshock and Pentylenetetrazole-Induced Seizures in Albino Mice. Int. J. Green. Pharm. 6, 63–66. 10.4103/0973-8258.97132

[B81] Patsoukis N. Zervoudakis G. Georgiou C. D. Angelatou F. Matsokis N. A. Panagopoulos N. T. (2005). Thiol Redox State and Lipid and Protein Oxidation in the Mouse Striatum after Pentylenetetrazol-Induced Epileptic Seizure. Epilepsia 46, 1205–1211. 10.1111/j.1528-1167.2005.63704.x 16060929

[B82] Perry N. S. Houghton P. J. Theobald A. Jenner P. Perry E. K. (2000). *In-vitro* Inhibition of Human Erythrocyte Acetylcholinesterase by *Salvia lavandulaefolia* Essential Oil and Constituent Terpenes. J. Pharm. Pharmacol. 52, 895–902. 10.1211/0022357001774598 10933142

[B83] Pitchaiah G. Viswanatha G. L. Nandakumar K. (2008). Pharmacological Evaluation of Alcoholic Extract of Stem Bark of *Erythrina variegata* for Anxiolytic and Anticonvulsant Activity in Mice. Pharmacol. Online 3, 934–947.

[B84] Porsolt R. D. Anton G. Blavet N. Jalfre M. (1978). Behavioural Despair in Rats: a New Model Sensitive to Antidepressant Treatments. Eur. J. Pharmacol. 47, 379–391. 10.1016/0014-2999(78)90118-8 204499

[B85] Prut L. Belzung C. (2003). The Open Field as a Paradigm to Measure the Effects of Drugs on Anxiety-like Behaviors: a Review. Eur. J. Pharmacol. 463, 3–33. 10.1016/s0014-2999(03)01272-x 12600700

[B86] Ragsdale D. S. Avoli M. (1998). Sodium Channels as Molecular Targets for Antiepileptic Drugs. Brain Res. Brain Res. Rev. 26, 16–28. 10.1016/s0165-0173(97)00054-4 9600622

[B87] Rajendran R. Ambikar D. B. Khandare R. A. Sannapuri V. D. Vyawahare N. S. Clayton P. (2014). Nootropic Activity of *Caralluma fimbriata* Extract in Mice. FNS 05, 147–152. 10.4236/fns.2014.52019

[B88] Ramanjaneyulu R. Ticku M. K. (1984). Interactions of Pentamethylenetetrazole and Tetrazole Analogues with the Picrotoxinin Site of the Benzodiazepine-GABA Receptor-Ionophore Complex. Eur. J. Pharmacol. 98, 337–345. 10.1016/0014-2999(84)90282-6 6327331

[B89] Rantakallio P. Leskinen M. von Wendt L. (1986). Incidence and Prognosis of central Nervous System Infections in a Birth Cohort of 12,000 Children. Scand. J. Infect. Dis. 18, 287–294. 10.3109/00365548609032339 3764348

[B90] Rasilingam D. Duraisamy S. Subramanian R. (2009). Anticonvulsant Activity of Bioflavonoid Gossypin. Bangladesh J. Pharmacol. 4, 51–54. 10.3329/bjp.v4i1.1081

[B91] Rauca C. Wiswedel I. Zerbe R. Keilhoff G. Krug M. (2004). The Role of Superoxide Dismutase and Alpha-Tocopherol in the Development of Seizures and Kindling Induced by Pentylenetetrazol - Influence of the Radical Scavenger Alpha-Phenyl-N-Tert-Butyl Nitrone. Brain Res. 1009, 203–212. 10.1016/j.brainres.2004.01.082 15120598

[B92] Ren Y. Houghton P. J. Hider R. C. Howes M. J. (2004). Novel Diterpenoid Acetylcholinesterase Inhibitors from *Salvia miltiorhiza* . Planta Med. 70, 201–204. 10.1055/s-2004-815535 15114495

[B93] Rocha L. Ackermann R. F. Engel J. (1996). Chronic and Single Administration of Pentylenetetrazol Modifies Benzodiazepine Receptor-Binding: an Autoradiographic Study. Epilepsy Res. 24, 65–72. 10.1016/0920-1211(95)00104-2 8796354

[B94] Rogawaski M. A. Porter R. J. (1990). Antiepileptic Drugs: Pharmacological Mechanisms and Clinical Efficiency with Consideration of Promising Developmental Stage Compounds. Pharmacol. Rev. 42, 233–286. 2217531

[B95] Rogers S. J. Cavazos J. E. (2008). “Epilepsy,” in Pharmacotherapy: A Pathophysiologic Approach. Editors DiPiro J. T. Talbert R. L. Yee G. C. Matzke G. R. Wells B. G. Posey L. M. 7th edn (New York: McGraw-Hill Co. Inc.), 927–951.

[B96] Rogóz Z. Skuza G. Kłodzińska A. (2003). Anxiolytic-like Effects of Preferential Dopamine D3 Receptor Agonists in an Animal Model. Pol. J. Pharmacol. 55, 449–454. 14506325

[B97] Salzberg M. Kumar G. Supit L. Jones N. C. Morris M. J. Rees S. (2007). Early Postnatal Stress Confers Enduring Vulnerability to Limbic Epileptogenesis. Epilepsia 48, 2079–2085. 10.1111/j.1528-1167.2007.01246.x 17999745

[B98] Samokhina E. Samokhin A. (2018). Neuropathological Profile of the Pentylenetetrazol (PTZ) Kindling Model. Int. J. Neurosci. 128, 1086–1096. 10.1080/00207454.2018.1481064 29792126

[B99] Santosh P. Venugopl R. Nilakash A. S. Kunjbihari S. Mangala L. (2011). Antidepressant Activity of Methanolic Extract of *Passiflora foetida* Leaves in Mice. Int. J. Pharm. Pharm. Sci. 3, 112–115.

[B100] Scholfield C. N. (1982). Antagonism of Gamma-Aminobutyric Acid and Muscimol by Picrotoxin, Bicuculline, Strychnine, Bemegride, Leptazol, D-Tubocurarine and Theophylline in the Isolated Olfactory Cortex. Naunyn Schmiedebergs Arch. Pharmacol. 318, 274–280. 10.1007/BF00501165 7078662

[B101] Schünzel G. Wolf G. Pomrenke U. Pomrenke C. Schmidt W. (1992). Pentylenetetrazol Kindling and Factors of Glutamate Transmitter Metabolism in Rat hippocampus. Neuroscience 49, 365–371. 10.1016/0306-4522(92)90102-8 1359455

[B102] Shakeri A. Sahebkar A. Javadi B. (2016). *Melissa officinalis* L. - A Review of its Traditional Uses, Phytochemistry and Pharmacology. J. Ethnopharmacol. 188, 204–228. 10.1016/j.jep.2016.05.010 27167460

[B103] Silambujanaki P. Chitra V. Kumari S. Sankari M. Raju D. Bala T. (2010). Anti-convulsant Activity of Methanolic Extract of *Butea monosperma* Leaves. Res. J. Pharm. Biol. Chem. Sci. 1, 431–435.

[B104] Singh D. Mishra A. Goel R. K. (2013). Effect of Saponin Fraction from *Ficus religiosa* on Memory Deficit, and Behavioral and Biochemical Impairments in Pentylenetetrazol Kindled Mice. Epilepsy Behav. 27, 206–211. 10.1016/j.yebeh.2012.11.004 23332444

[B105] Steru L. Chermat R. Thierry B. Simon P. (1985). The Tail Suspension Test: a New Method for Screening Antidepressants in Mice. Psychopharmacology (Berl) 85, 367–370. 10.1007/BF00428203 3923523

[B106] Storm J. F. (1987). Action Potential Repolarization and a Fast After-Hyperpolarization in Rat Hippocampal Pyramidal Cells. J. Physiol. 385, 733–759. 10.1113/jphysiol.1987.sp016517 2443676 PMC1192370

[B107] Taiwe G. S. Moto F. C. Ayissi E. R. Ngoupaye G. T. Njapdounke J. S. Nkantchoua G. C. (2015). Effects of a Lyophilized Aqueous Extract of *Feretia apodanthera* Del. (Rubiaceae) on Pentylenetetrazole-Induced Kindling, Oxidative Stress, and Cognitive Impairment in Mice. Epilepsy Behav. 43, 100–108. 10.1016/j.yebeh.2014.11.022 25601583

[B108] Thesleff S. (1980). Aminopyridines and Synaptic Transmission. Neuroscience 5, 1413–1419. 10.1016/0306-4522(80)90002-0 6250099

[B109] Turhan M. (2006). Hand Book of Herbal Plants, Chapter 4. Melissa officinalis 3, 184–245.

[B110] Uva L. Trombin F. Carriero G. Avoli M. de Curtis M. (2013). Seizure-like Discharges Induced by 4-aminopyridine in the Olfactory System of the *In Vitro* Isolated guinea Pig Brain. Epilepsia 54, 605–615. 10.1111/epi.12133 23505998 PMC4891192

[B111] Velísek L. Vachová D. Mares P. (1997). Excitatory Amino Acid Antagonists and Pentylenetetrazol-Induced Seizures during Ontogenesis. IV. Effects of CGP 39551. Pharmacol. Biochem. Behav. 56, 493–498. 10.1016/s0091-3057(96)00245-6 9077588

[B112] White H. S. (1997). “New Mechanisms of Antiepileptic Drugs II,” in Epilepsies. Editors Porter R. Chadwick D. (Boston: Butterworth Heinemann), 1–30.

