# Supplementary figures and images for: New Insights Into the Anticonvulsant Effects of Essential Oil From Melissa officinalis L. (Lemon Balm)
Source: Front Pharmacol. 2021 Oct 14;12:760674. doi: 10.3389/fphar.2021.760674 (PMC8551917; doi:10.3389/fphar.2021.760674)

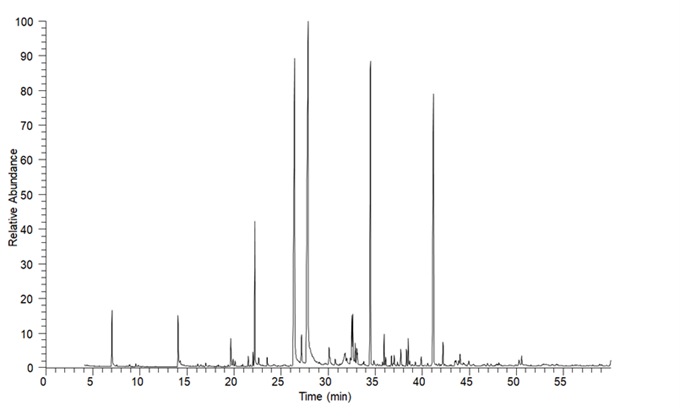

Supplement: Supplementary file 1 [file Image1.png]
